# Asymmetric Electrolytes Design for Aqueous Multivalent Metal Ion Batteries

**DOI:** 10.1007/s40820-023-01256-6

**Published:** 2023-12-15

**Authors:** Xiaochen Yang, Xinyu Wang, Yue Xiang, Longtao Ma, Wei Huang

**Affiliations:** 1 Frontiers Science Center for Flexible Electronics, Institute of Flexible Electronics, Northwestern Polytechnical University, Xi’an, 710072 People’s Republic of China; 2 School of Materials Science and Engineering, Guangdong Provincial Key Laboratory of Advanced Energy Storage Materials, South China University of Technology, Guangzhou, 510641 People’s Republic of China

**Keywords:** Asymmetric electrolyte, Aqueous multivalent metal ion batteries, Electrochemical stability windows, Electrolyte interface

## Abstract

With the rapid development of portable electronics and electric road vehicles, high-energy-density batteries have been becoming front-burner issues. Traditionally, homogeneous electrolyte cannot simultaneously meet diametrically opposed demands of high-potential cathode and low-potential anode, which are essential for high-voltage batteries. Meanwhile, homogeneous electrolyte is difficult to achieve bi- or multi-functions to meet different requirements of electrodes. In comparison, the asymmetric electrolyte with bi- or multi-layer disparate components can satisfy distinct requirements by playing different roles of each electrolyte layer and meanwhile compensates weakness of individual electrolyte. Consequently, the asymmetric electrolyte can not only suppress by-product sedimentation and continuous electrolyte decomposition at the anode while preserving active substances at the cathode for high-voltage batteries with long cyclic lifespan. In this review, we comprehensively divide asymmetric electrolytes into three categories: decoupled liquid-state electrolytes, bi-phase solid/liquid electrolytes and decoupled asymmetric solid-state electrolytes. The design principles, reaction mechanism and mutual compatibility are also studied, respectively. Finally, we provide a comprehensive vision for the simplification of structure to reduce costs and increase device energy density, and the optimization of solvation structure at anolyte/catholyte interface to realize fast ion transport kinetics.

## Highlights

- The working principle of the asymmetric electrolyte and the long-term-seated contradictory issues were analyzed.
- The characterization methods for the interfaces of anolyte/catholyte and electrolyte/electrode were summarized for revealing the fundamental mechanism of asymmetric electrolytes.
- The future research directions for asymmetric electrolyte systems were proposed.

## Graphical abstract

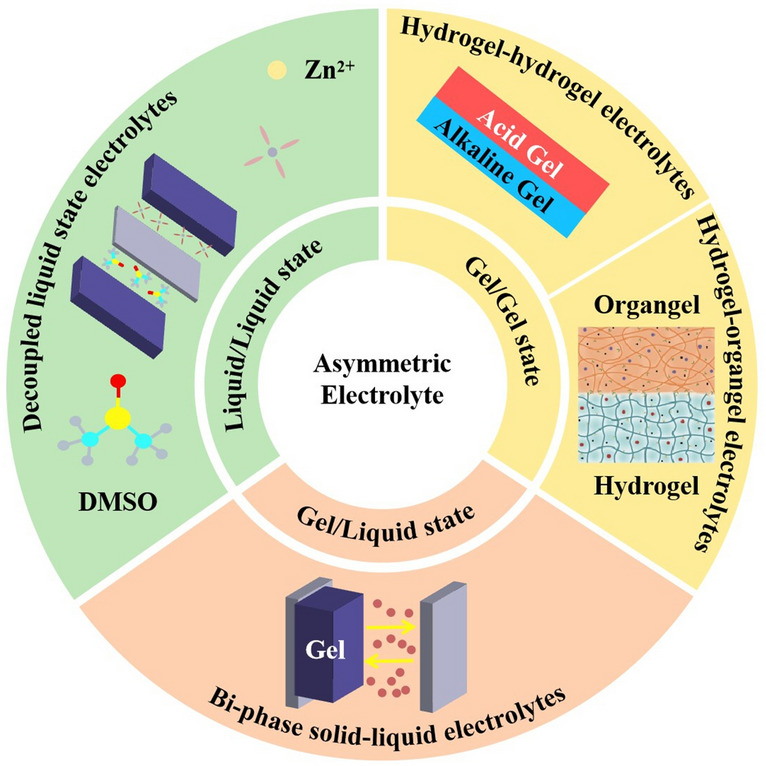

## Introduction

**Fig. 1:**
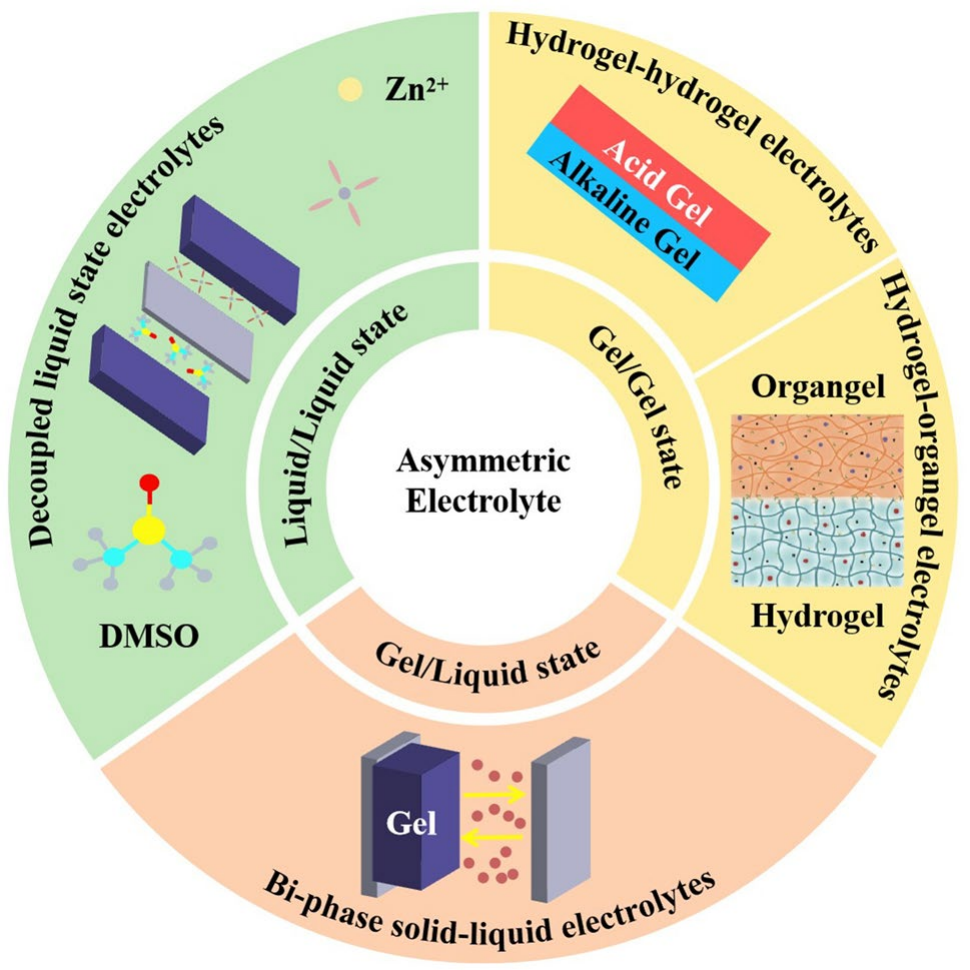
Aqueous metal batteries (AMB) overview and classification of asymmetric electrolyte strategies, including liquid/liquid state, gel/liquid state and gel/gel state

**Fig. 2:**
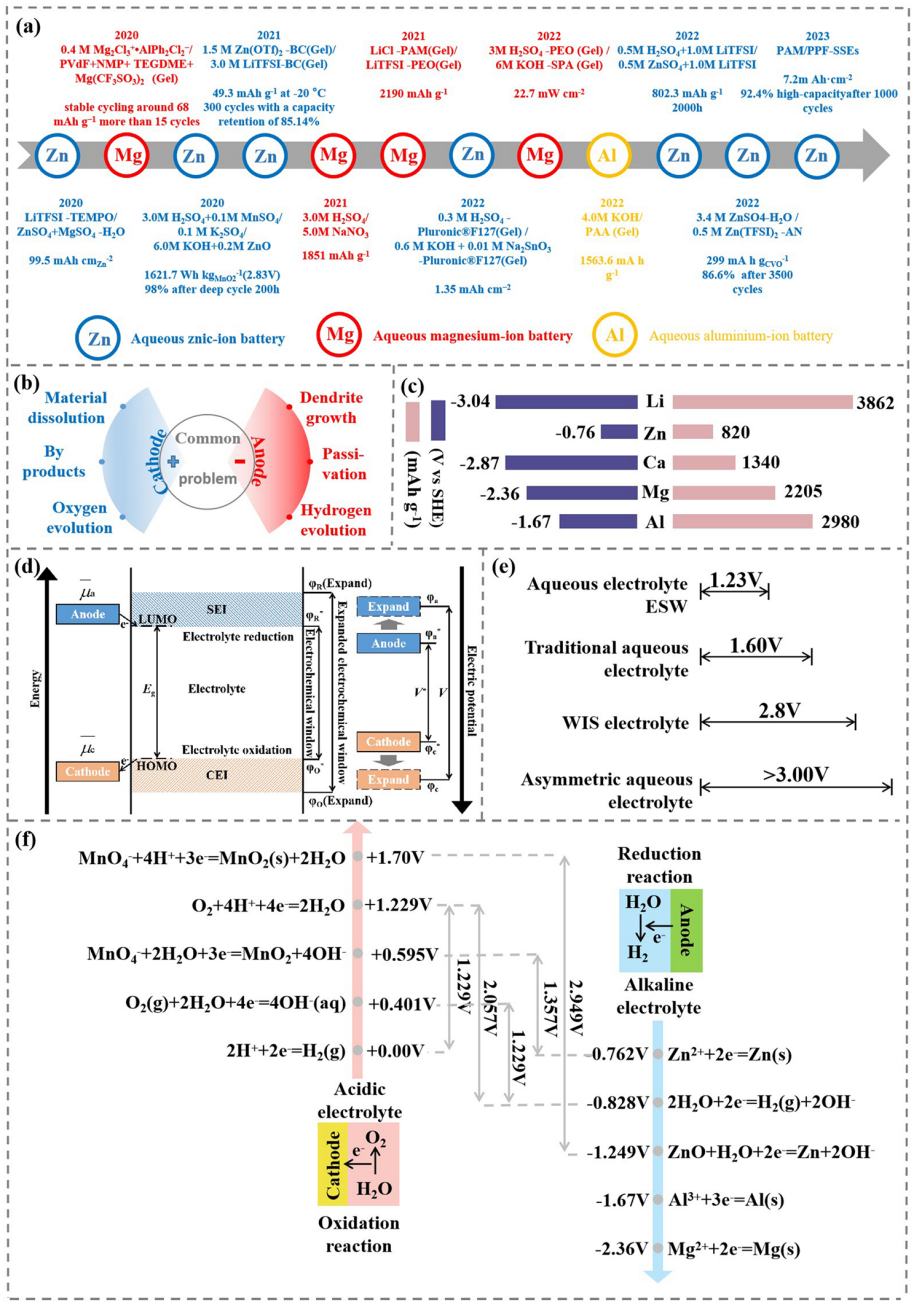
**a** Development history of asymmetric electrolyte in AMB [24, 25, 34–45]. **b** Common problems of AMB batteries in cathode and anode. **c** Reduction potential and specific capacity of several common metal carriers in AMB (blue is potential(V vs. SHE), pink is specific capacity (mAh g.^−1^)) [46]. **d** Fermi level schematic and electrochemical reactions of various elements under acidic or alkaline conditions [30, 46]. **e** ESW comparison of various aqueous electrolyte [47]. **f** The potential of redox reaction with different electrolytes in AMB [24, 25, 34–44]

Clean energy sources such as wind, solar and tidal power have grown rapidly in recent years with carbon–neutral targets [1]. These clean energy sources are volatile due to the effects of climate and cannot meet people’s need for a consistent output of energy [2, 3]. Therefore, it is necessary to develop large-scale energy storage equipment with high energy density, long cycle life and low cost [4–6]. Lithium-ion batteries (LIB) are widely used in electric vehicles and mobile electronic devices [7] and have made great progress in energy density and cycle life [8]. However, the increasing cost caused by resource shortage and safety issues in organic electrolyte systems have always limited the application of LIB in the field of large-scale energy storage and wearable devices [9–11]. Therefore, there is a high demand for the development of a new generation of energy storage equipment with high safety, environmental friendliness and rich raw material storage [12–16]. The utilization of water as solvent is a kind of aqueous electrolyte. Meanwhile, the Zn^2+^, Mg^2+^, Al^3+^ and other polyvalent AMB have high theoretical capacity, high safety and abundant reserves in the Earth’s crust, which are considered to have great application prospects [17–23]. In order to pursue higher ESW, longer cycle times and higher energy density, asymmetrical electrolytes have been developed. Since 2020, asymmetric electrolytes have gradually attracted more attention from scholars. In 2020, Hu et al. used H_2_SO_4_ as catholyte and KOH as anolyte to make the Zn/Mn battery system reach a high voltage of 2.83 V through acid–base decoupling electrolyte system, and the assembled 3.33 Ah battery pack reached a high energy density of 90 Wh kg_cell_^−1^ [24]. The energy density of the battery is higher than that of the traditional lead-acid battery, and the generation cost is lower, so it has a broad application prospect in the commercial field (Fig. 1). In 2021, Zhi et al. formed a highly flexible Zn-Air battery by Pluronic®F127 gel system using acid gel as catholyte and alkaline gel as anolyte [25]. The battery has high flexibility and a high current density of 1.35 mAh cm^−2^, providing new possibilities for wearable batteries. Asymmetric electrolytes have a lot of research results in aqueous Zn ion batteries (AZIB), and there are also a lot of research progress in Mg and Al batteries. The development roadmap of AMB using asymmetric electrolyte is shown in Fig. 2a.

Although more research has been done on AMB, problems such as dendrite growth, passivation and corrosion, hydrogen evolution reaction (HER) can seriously lower reversibility of metal anode and lead to short circuit with safety problems triggered [26]. At the cathode side, electrode materials dissolution and by-products formation will shorten battery lifespan and bring about electrode degradation (Fig. 2b) [27]. Traditional homogeneous electrolytes are impossible to simultaneously solve the issues of cathode and anode, and also difficult to meet the redox requirements of cathode and anode [28]. This review summarizes some asymmetric electrolyte systems in multivalent metal ion batteries. The theoretical capacity and reduction potential of some common metal ion batteries are shown in Fig. 2c.

In AMB systems, water is employed as the solvent for the electrolyte, which is limited by hydrogen evolution oxygen evolution (HER) and oxygen evolution (OER) potentials in a single electrolyte environment. In theory, aqueous electrolyte only shows electrochemical stability windows (ESW) of 1.23 V [29]. To stabilize cathode and anode, the operating voltage of AMB is limited between φ_R_* and φ_O_*, in which the *E*_*g*_ is 1.229 eV (Fig. 2d). However, in practice, anodes with higher Fermi level can be selected, because their Fermi level is higher than the lowest unoccupied molecular orbital (LUMO) of the electrolyte. Similarly, cathode with lower Fermi level can also be chosen because its Fermi level is lower than the highest occupied molecular orbital (HOMO) of the electrolyte [30]. To achieve excellent cyclic stability, anodes provide electrons to anolyte for the generation of solid electrolyte interface (SEI) by partial decomposition of electrolyte components. The SEI layer will change the state of the interface between anodes and anolyte, which further affects the work function and weakens capability of LUMO to receive electrons. Consequently, electrons do not have sufficient dynamic conditions to occupy LUMO and tend to flow to the external circuit [31]. Likewise, the cathode obtains electrons from the catholyte for the formation of cathode electrolyte interface (CEI) by oxidizing electrolyte. As a result, the electron transfer ability between cathode and the HOMO of electrolyte is weakened, ultimately resulting in insufficient dynamic conditions for electrons to escape from the HOMO. The cathode begins to accept electrons from outer circuit, resuming normal battery behavior. Although Fermi level can be used to explain the principle of electron transition during charge/discharge process, Fermi level cannot account for the substance involved in the redox reaction. The redox potential is closely related to the Gibbs free energy difference between reactants and products. Thus, the stability between electrolyte and electrode cannot be explained simply by Fermi level [30]. Ideally, the SEI and CEI are usually ionic conductors that prevent further electrochemical reactions between the electrodes and the electrolyte. The ESW of electrolyte is expanded from V^*^ to V [32]. Although AMB can broaden ESW by generating SEI and CEI, conventional electrolytes can only increase the voltage window to a small extent (Fig. 2e). The WIS electrolyte can improve the ESW of the aqueous battery by changing the solvation structure of metal cation and water. In traditional dilute aqueous solution electrolytes, metal cations and multiple water molecules form stable solvation structures. However, the concentration of salt in WIS electrolytes is so high that there are not enough water molecules and metal cations to form a solvation structure. Thus, the salt and metal cation form a solvation structure, and a small number of water molecules in the electrolyte are also surrounded by salt [33]. This also reduces the chance of contact between water molecules and the metal anode, thus reducing the corrosion of the electrode by water molecules. It can be concluded that the WIS electrolyte expands the ESW of the battery by extending the Fermi level of the electrolyte. However, expanding the Fermi level of the electrolyte is limited to expanding the ESW of the battery. The asymmetric electrolyte system can make the cathode and anode of the battery redox reaction under different electrolyte system. So we can change the redox reaction of the anode and cathode by designing different anolyte and catholyte. Thus, the Fermi levels of the cathode and anode are changed, which can effectively expand the ESW of the battery.

The asymmetric electrolytes are formed by assembling different catholyte and anolyte, which can meet contradictory requirements of cathode and anode. Decoupled electrolytes paired with acidic catholyte and alkaline anolyte can achieve an ESW of 2.057 V, which is much wider than theoretical ESW of 1.23 V in a single alkaline or acidic electrolyte. (Fig. 2f). In ZnǀǀMn battery systems, the adjustable ESW extends to 2.949 V with decoupled asymmetric electrolyte, much higher than the 1.357 V of conventional alkaline ZnǀǀMn batteries. Asymmetric electrolyte enables AMB to have a higher ESW, solving the bottleneck lower voltage of traditional AMB.

This review focuses on the research progress of asymmetric electrolytes, including the preparation process and working principle the advantages existing problems. After that, the application prospect of asymmetric electrolytes in flexible energy storage devices and large-scale power grid energy storage is forecasted.

## Feature and Superiority of Asymmetric Electrolyte

Although Zn, Mg, Al and Ca are considered to be very promising for AMB, due to their low toxicity, high safety and low cost, various issues exist in the cathode and anode of AMB. Polyvalent metals can transport more electrons than alkaline metal, which hopefully provides high capacity. However, the polyvalent metal ions usually hydrate with water molecules at the cathode due to their strong polarization reaction, leading to a larger cage structure (*e.g.*, Zn^2+^ forms a cage structure with a size of 5.5 × 10^–10^), which also leads to the difficulty in the insertion/withdrawal of polyvalent metal ions at the cathode and thus slow ion transfer dynamics [48, 49].

At the anode of AMB, the formation of alkaline conditions resulting from hydrogen production and thermodynamic instability of polyvalent metal in alkaline environment lead to the gradual generation of metal hydroxide or oxide, which seriously deteriorates the reversibility of plating/stripping process. At the cathode of AMB, OER usually occurs at high potential, and the resulting gas causes the pressure inside the battery to increase, and the bubbles also reduce the rate of ion conduction. CEI is generated locally in the process of battery charge–discharge cycle, but in situ CEI is usually unstable and cannot prevent cathode and electrolyte from continuously reacting. The dissolution of active substances in cathode and the formation of by-products are one of the important reasons for the decline of the capacity and long cycle properties of AMB [50–52].

In aqueous multivalent metal ion batteries, the cathode and anode of the battery face different problems, respectively. For example, anode has dendrite growth, passivation and corrosion, HER and other problems. There are electrode materials dissolution, by-products and OER and other problems in the cathode. The traditional single electrolyte cannot solve the problems of cathode and anode at the same time, and it is contradictory to meet the rapid dynamic demand of cathode and the thermodynamic stability of anode at the same time. However, the asymmetric electrolyte system can choose a variety of different electrolytes to meet the needs of the cathode and anode, respectively. This allows the battery to have fewer side reactions, resulting in a longer cycle life, higher capacity and power density. At the same time, for aqueous batteries, the ESW of water-based batteries is difficult to reach 2 V due to the decomposition potential of water (~ 1.23 V) [53]. Asymmetric electrolytes can be used to broaden the ESW of the battery more effectively by changing the pH of the aqueous electrolyte or selecting organic electrolyte. Therefore, broadening the ESW of the battery can directly improve the energy density of the battery, and at the same time, the battery has a broader application space.

**Fig. 3:**
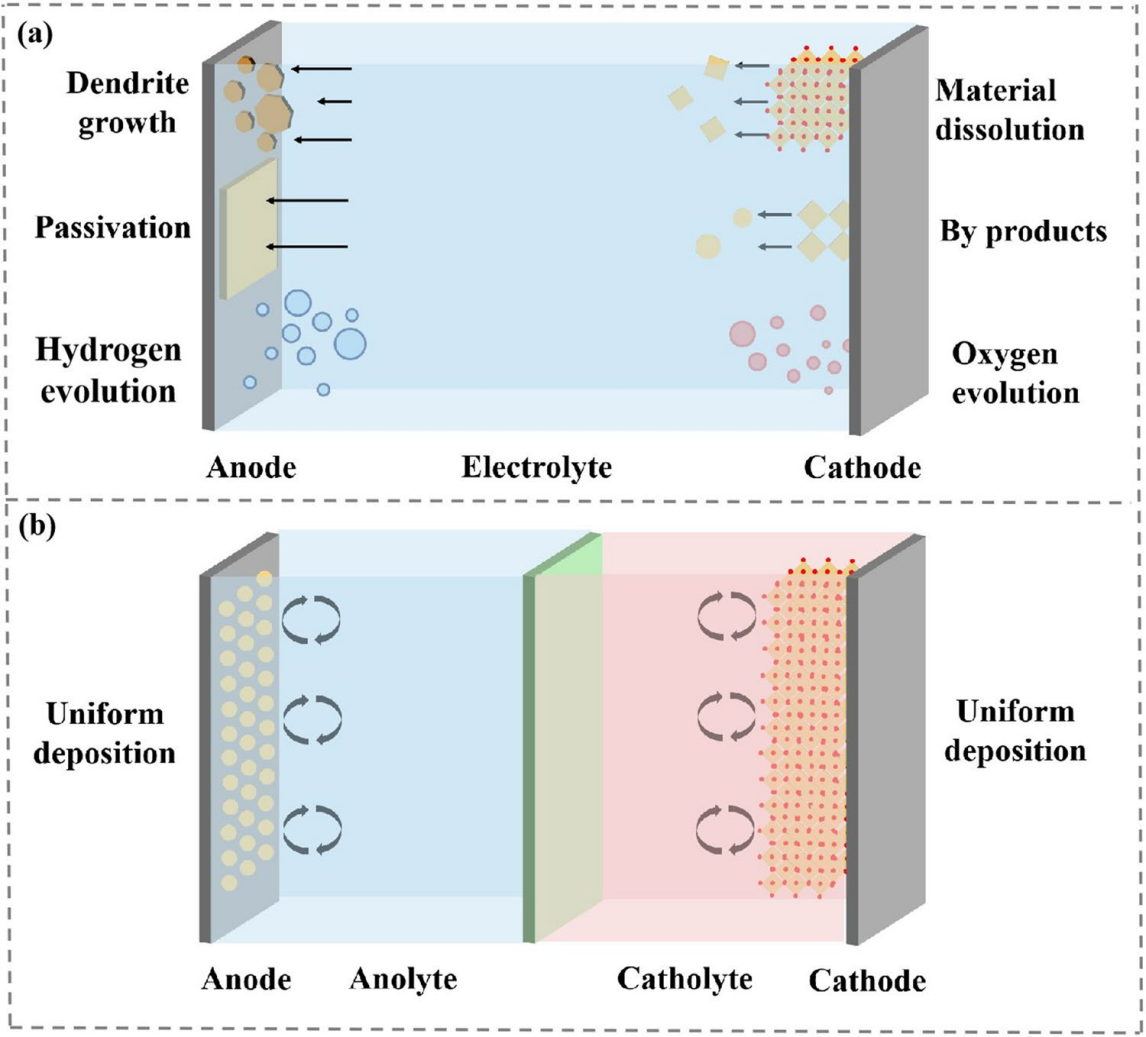
Comparison of conventional homogeneous electrolyte and asymmetric electrolyte. **a** Common problems of drainage batteries in cathode and anode. **b** Asymmetrical electrolytes satisfy both cathode and anode requirements

Asymmetric electrolytes possess two or more-layer different electrolytes, which allows the catholyte and the anolyte to solve issues at the cathode and anode interface, respectively (Fig. 3b).

**Fig. 4:**
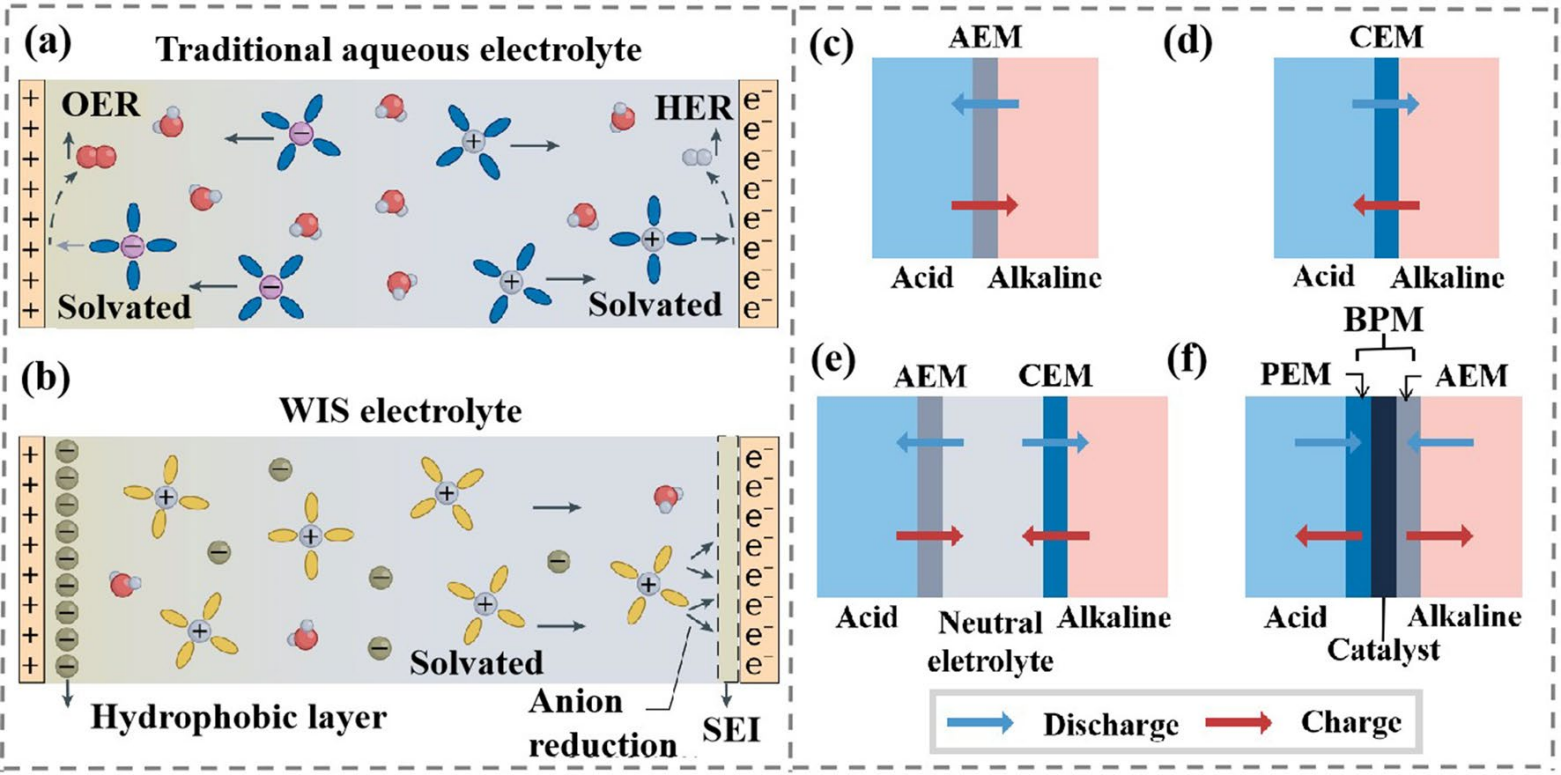
**a** Water hydrolyzes in conventional electrolytes. Copyright 2015, Nature Publishing Group [60]. **b** Water hydrolysis is inhibited in the WIS electrolyte. Copyright 2022, Nature Publishing Group [60]. **c-f** Four typical membrane structures in AMB. Copyright 2022, Nature Publishing Group [60]

In addition, the output voltage of AMB is limited to stable voltage window of water (~ 1.23 V). A large number of water molecules in AMB forms hydrogen bond networks, weakening the strength of O–H covalent bonds [54], which promotes high reactivity of water molecules. Water hydrolysis in conventional aqueous electrolytes is shown in Fig. 4a. When the charging voltage is higher than the electrochemical window of AMB, water molecules undergo oxygen evolution reactions (OER) and HER at the cathode and anode, respectively [55]. In order to suppress the participation of water decomposition, highly concentrated hydrophobic ions (such as TFSI^−^ and OTf^−^) are added into water solvent develop a concentrated “water in salt (WIS)” electrolyte. Meanwhile, abundant hydrophobic anions gather on the cathode side, which is repulsive to water from cathode and effectively alleviates the OER at the cathode/electrolyte interface [56]. On the other hand, a stable SEI film with electronic insulation and ionic conductivity properties at the anode is formed by reducing F^−^ contained anions, which effectively suppress HER and thus further broadens the electrochemical window of AMB (Fig. 4b).

In comparison with homogeneous electrolyte, decoupled liquid-state electrolytes are composed of acidic electrolytes as catholyte and alkaline electrolytes as anolyte. For instance, the standard reduction potential of Zn/Zn^2+^ in neutral electrolytes is -0.76 V vs. SHE, while that of Zn/ZnO in alkaline electrolytes is -1.249 V vs. SHE, which potentially increases the battery voltage by 0.5 V. The standard reduction potential of MnO_4_^−^/MnO_2_ in alkaline electrolytes is 0.595 V vs. SHE, while the standard reduction potential of MnO_4_^−^/MnO_2_ (s) in acidic electrolytes can reach 1.70 V vs. SHE, which increases the potential of the cathode by nearly 1.1 V.

It is noted that acidic catholyte and basic anolyte are prone to neutralization reactions, so ion-selective membranes (IEM) are needed to inhibit the chemical reactions of H^+^ and OH^−^. IEM is divided into cation-exchange membranes (CEM) and anion-exchange membranes (AEM) [57]. Usually composed of hydrophobic polymer substrates and fixed ion-functionalized groups, IEM conducts ion conduction through ion concentration gradients and potential gradients [58]. IEM is easy to be corroded in strong acid and base environment and cannot effectively prevent the chemical reaction of H^+^ and OH^−^ on both sides of IEM during long-term storage. Therefore, neutral electrolyte is added to catholyte and anolyte. This system further separates acid catholyte from alkaline anolyte and effectively prevents H^+^ from reacting with OH^−^. This is coupled with a bipolar membrane (BPM) that effectively insulates anolyte and catholyte [59]. BPM consists of a proton-exchange membrane (PEM) and an AEM. The PEM and AEM are mixed with catalysts that can hydrolyze H_2_O to H^+^ and OH^−^, which can appropriately replenish the consumed H^+^ and OH^−^. Four typical membrane structures are shown in Fig. 4c-f.

## Classification of Asymmetric Electrolytes

In this review, asymmetric electrolytes are divided into three types in terms of physical state: decoupled liquid-state electrolytes, bi-phase solid/liquid electrolytes and asymmetric quasi-solid-state electrolytes. In the field of large-scale energy storage, decoupled liquid-state electrolytes and bi-phase solid–liquid electrolytes have broad application prospects because of their low cost, high safety, high energy density and long cycle life. Traditional aqueous metal ion batteries usually have various side reactions that lead to short battery cycle life, which leads to their limited application in the field of large-scale energy storage. However, in a system of decoupled liquid-state electrolytes and bi-phase solid–liquid electrolytes, by choosing organic electrolytes as anolyte. It can prevent the corrosion of Zn anode by water, thus effectively extending the life of the battery. At the same time, the change of the REDOX reaction of the asymmetric electrolyte to the electrode also effectively expands the voltage of the battery, thus increasing the energy density of the battery. Therefore, asymmetric electrolytes can help aqueous multivalent metal ion batteries become more competitive in the field of large-scale energy storage. In the field of wearable flexible batteries, aqueous metal ion batteries have a wide range of application prospects because of their high flexibility, high safety and non-toxicity. However, the voltage of a water cell is limited by the decomposition potential of water (~ 1.23 V), which leads to limited application and energy density of a single cell. Asymmetric quasi-solid-state electrolyte maintains the safety of aqueous battery and has better performance in inhibiting dendrites and HER, so that the battery has longer cycle life and higher capacity. Gel electrolytes are usually rich in hydrophilic functional groups (such as -OH, -COOH and -NH_2_), which can change the hydrogen bonding force before the water molecules in the aqueous electrolyte, thereby improving the low-temperature performance of the battery. The asymmetric quasi-solid-state electrolyte can be applied in a wide range of temperature conditions. Same as decoupled liquid-state electrolytes and bi-phase solid–liquid electrolytes, asymmetric quasi-solid-state electrolyte can also effectively expand the voltage of a single battery, which makes aqueous multivalent metal ion batteries have a broader application prospect in the field of wearable flexible batteries.

In this section, we commit to establish an accessible athenaeum of the physicochemical characterizations of diversified asymmetric electrolytes and their electrochemical possibilities.

### Decoupled Liquid-State Electrolytes

In traditional battery systems, both the reduction reaction at the cathode and the oxidation reaction at the anode during discharging process are in same electrolyte environment. Decoupled liquid-state electrolytes consist of two different kinds of catholyte and anolyte, in which catholyte provides an electrochemical reaction environment for cathode reduction reactions while anolyte provides another district for the oxidation of anodes. According to species and electrochemical properties of catholyte and anolyte, decoupled liquid-state electrolytes are divided into bi-phase aqueous/organic liquid electrolytes and PH-decoupled dual-liquid-state electrolytes.

#### Bi-phase Aqueous/Organic Liquid Electrolyte

Aqueous zinc-ion batteries have been widely studied for their advantages of safety, non-toxicity and low cost. In aqueous electrolytes, the solvation structure of Zn^2+^ and water molecules has fast transfer and deposition kinetics. This gives the battery a high power density. However, problems such as hydrogen evolution reaction and dendrite growth in aqueous battery also seriously affect the cycle life of aqueous battery. Compared with aqueous electrolyte, zinc is easily deposited along the (101) crystal face, resulting in dendrite growth. In the organic electrolyte, the organic electrolyte can promote the growth of zinc along the (002) crystal surface due to its slow kinetics, avoiding the problems of “dead zinc” and dendrite growth [61]. Meanwhile, the organic electrolyte also completely avoids the corrosion of water molecules on the electrode and avoids side reactions such as hydrogen evolution. Therefore, the organic electrolyte allows the battery to have a longer cycle life and a higher capacity retention rate. Organic electrolytes also perform better than aqueous electrolytes at low temperatures because of their lower melting point [62, 63]. With aqueous electrolytes, the delivered capacity and cycling stability of the AMB are seriously affected by HER and metal anode corrosion. The utilization of organic electrolyte is proposed as an effective route to protect the anode while maintaining a certain unit power capacity. However, organic electrolyte tends to destroy the cathode structure, resulting in a decrease in battery capacity. At high current density, the organic electrolyte slow reaction kinetics cannot meet the demand of cathode. Therefore, it is usually necessary to design an asymmetric electrolyte system with aqueous electrolyte as the cathode electrolyte and organic electrolyte as the anode electrolyte to achieve higher capacity, higher power density and longer long cycle performance. The selection of appropriate organic electrolytes can isolate the zinc anode from the aqueous electrolyte, thereby increasing the cycle capacity of the battery. In aqueous electrolyte system, the potential of zinc anode is limited by the hydrogen evolution potential of water. However, when the organic electrolyte acts as anolyte, the potential of the zinc anode will not be limited by the hydrogen evolution potential of water. Therefore, the Fermi level of the zinc anode is greatly expanded, which also further expands the ESW of the battery.

Non-membrane two-liquid electrolytes have attracted much attention because of their high ionic conductivity and high energy density. Aqueous electrolytes are selected as catholyte and hydrophobic organic electrolytes as anolyte. Due to their thermodynamic properties and polarity differences, the bi-phase electrolytes are immiscible. Bi-phase aqueous/organic liquid electrolyte showed much higher discharge capacity and long cycle capacity than traditional AMB.

**Fig. 5:**
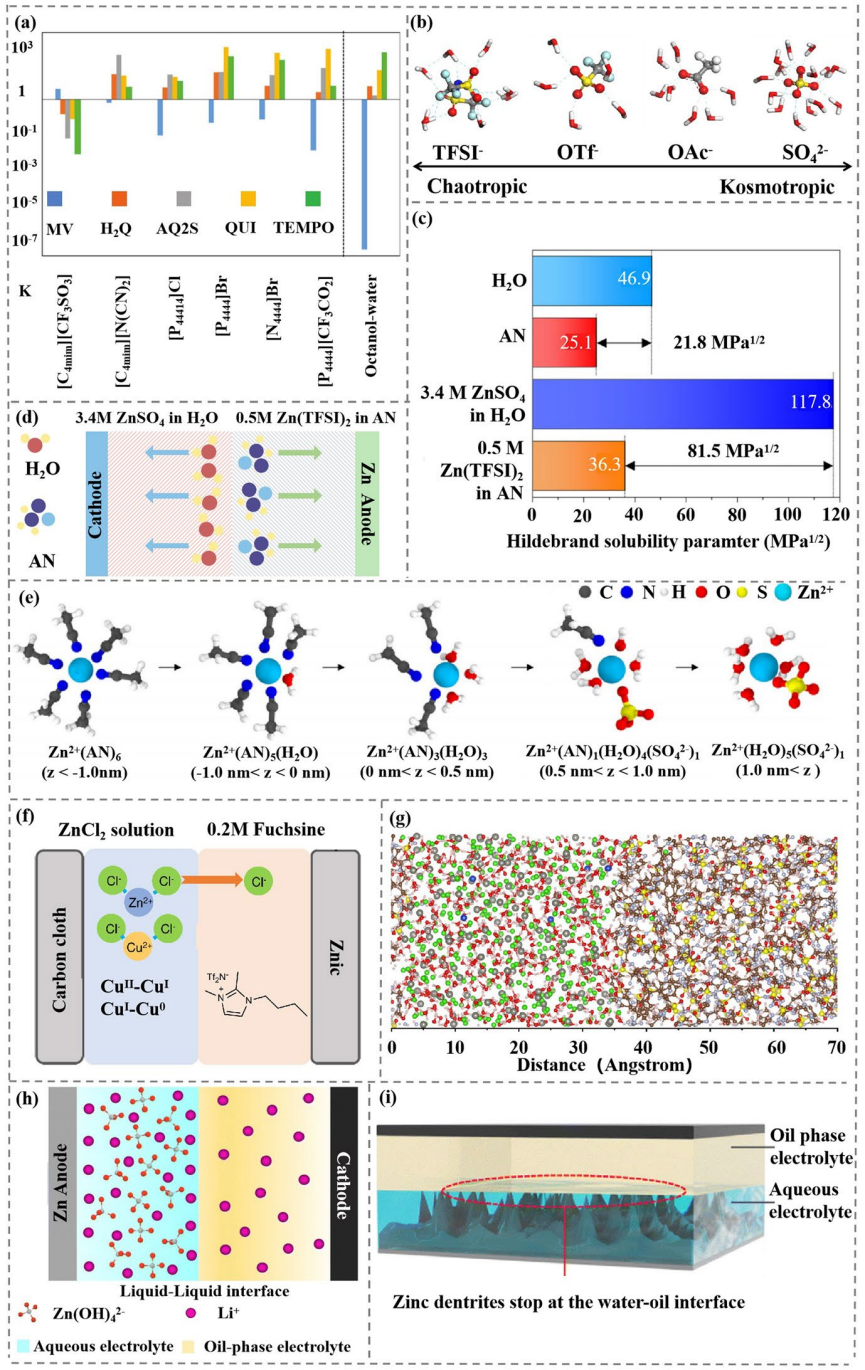
**a** Partition coefficients (K = [molecule]_top phase_/[molecule]_bottom phase_) of the target molecules in ABS based on different ionic liquids. Copyright 2018, Wiley–VCH [41]. **b** Different anions produce different hydration structure by chaotropic and kosmotropic effect. Gray, blue, white, red, yellow and cyan balls represent carbon, nitrogen, hydrogen, oxygen, sulfur and fluorine, respectively. Copyright 2022, The Royal Society of Chemistry [36]. **c** Hildebrand solubility parameter values of the solvents and electrolytes. D_d_ denotes the difference in the solubility parameter between the two components. Copyright 2022, The Royal Society of Chemistry [36]. **d** After the addition of SO_4_^2−^ in catholyte and TFSI^−^ in anolyte, the aqueous solvent and AN solvent repel each other and are immiscible. **e** Snapshots depicting the change in the first solvation shell of Zn^2+^ at OE-rich region (z < −1.0 nm), interface region (−1.0 nm < z < 1.0 nm) and AE-rich region (z > 1.0 nm). Copyright 2022, The Royal Society of Chemistry [36]. **f** Schematic illustration of the rechargeable Zn-Cu battery for the flexible cell configuration. Copyright 2023, Nature Publishing Group [67]. **g** A snapshot extracted from the simulation of the aqueous CuCl_2_-ZnCl_2_ solution/IL system. Copyright 2023, Nature Publishing Group [67]. **h** Schematic illustration of the PSE in zinc batteries. Copyright 2023, The Royal Society of Chemistry [72]. **i** Schematic illustration of Zn dendrite growth stopping at the interface. Copyright 2023, The Royal Society of Chemistry [72]

To solve the catholyte and anolyte problem simultaneously, attempts have been made to combine the appropriate catholyte and anolyte on the cathode and anode sides, respectively, to meet the redox requirements of both the cathode and the anode. REDOX molecules with quite different partition coefficients (K) of ionic liquids (IL) are selected as a non-membrane two-phase battery, which is assembled by the miscibility of the two redox molecules [41, 64]. In this two-liquid system, the top and bottom phases are composed of Na_2_SO_4_, IL and H_2_O (10, 35 and 55 wt%). REDOX molecules with K value higher than 1 are mainly used in the top phase, which is mostly IL-rich phase. The redox molecules with K value less than 1 are preferentially assigned to the salt-rich phase, which is the bottom phase (Fig. 5a). This work also proves that this aqueous non-membrane battery consisting of MV and TEMPO[P_44414_]Cl^−^ can be used at 1.35 V working voltage with high coulomb efficiency. Electron transport in the top and bottom phases of the battery system is achieved through TEMPO and MV REDOX reactions in which electrons are gained and lost in electrochemical reactions.

Immiscible biphasic liquid electrolyte is designed based on the kosmotropic effect and chaotropic effect of ions [36]. It is known that the water structure is more affected by anions than cations. Small ions with high charge density have strong Kosmotropic effect, which can strengthen the affinity of ions with H_2_O by strengthening the hydrogen bond in H_2_O. So, the ions are closely combined with H_2_O, and do not dissolve in other solvents [65]. On the contrary, large ions with low charge density have strong Chaotropic effect. By destroying the hydrogen bond in H_2_O, the binding force between ions and H_2_O phase is weak, and thus they do not dissolve in H_2_O phase (Fig. 5b). In this BLE, 3.4 M ZnSO_4_ in H_2_O is used for Catholyte and 0.5 M Zn (TFSI)_2_ in AN is used for Anolyte. After adding SO_4_^2−^ and TFSI^−^, H_2_O and AN are mutually exclusive due to kosmotropic effect and chaotropic effect [66], showing no miscibility. The solubility difference between ZnSO_4_ and Zn(TFSI)_2_ when added is shown in Fig. 5c. The organic electrolyte on the Zn anode side prevents the aqueous electrolyte from HER, passivation and Zn dendrite growth, while the water electrolyte at the cathode side provides the conditions for the rapid reaction kinetics of Zn^2+^ (Fig. 5d). The solvated structure of Zn^2+^ from organic electrolytes (OE) to aqueous electrolyte (AE) is changed as shown in Fig. 5e. In the OE phase (z < -1.0 nm), Zn^2+^ and AN formed the solvated structure of Zn^2+^(AN)_6_. At the interface of OE-AE (-1.0 < z < 1.0 nm) gradually desorbed AN molecule, and more H_2_O molecules are added to the solvated structure. In the AE phase (z > 1.0 nm), SO_4_^2−^ was added to the solvated structure to form a stable Zn^2+^(H_2_O)_5_(SO_4_^2−^)_1_ solvated structure [36]. Non-polar solvents electrolytes exhibit low ionic conductivity due to their insufficient dissociation capacity. However, polar solvent electrolytes have higher ionic conductivity. Then, the theoretical calculation shows that the ionic conductivity of the biphasic electrolyte is similar to that of the OE-AE interface resistance, which indicates that the OE-AE interface resistance can be ignored. The low resistance and activation energy of the OE-AE interface allow Zn^2+^ to pass through the interface quickly, thus improving the power density of the battery. Zn^2+^ gradually changes the solvation structure near the OE-AE interface. This process is very stable and highly reversible, which promote excellent cyclic stability and capacity retention. The full battery using the Zn anode and CaV_6_O_16_ (CVO) delivers a high specific capacity of 299 mAh g^−1^ and a long cyclic stability with 86.6% initial capacity retained after 3,600 cycles.

Flexible rechargeable Zn//Cu battery is developed based aqueous catholyte with CuCl_2_ and ZnCl_2_ and ionic organic liquid anolyte based on Tf_2_N (Fig. 5f) [67]. The hydrophobic Tf_2_N is selected to restrict Cu^2+^ in aqueous catholyte, and Cl^−^ is used as the charge carrier between the two phases to maintain the electric neutrality. In aqueous anolyte, in comparison with a distorted octahedral structure of Cu^2+^ with H_2_O and Cl^−^, 1 M CuCl_2_ combined with 15 M ZnCl_2_ form a specific solvation structure of CuCl_2_(H_2_O)_4_ [68–71].

It can be obviously observed that good delamination occurs at the interface of aqueous anolyte and organic catholyte (Fig. 5g). In The reduction of copper is divided into two steps, Cu^II^ → Cu^I^ → Cu^0^. The specific reaction chloride ion shuttle at the electrode can be expressed by Eqs. (1–3):

Cathode:

1 $$ { }\left[ {{\text{CuCl}}_{{\text{x}}} } \right]^{2 - x} + {\text{e}}^{ - } \to { }\left[ {{\text{CuCl}}} \right]_{x}^{1 - x} { }\left( {1.3\;{\text{V vs}}.{ }{{{\text{Zn}}} \mathord{\left/ {\vphantom {{{\text{Zn}}} {{\text{Zn}}}}} \right. \kern-0pt} {{\text{Zn}}}}^{2 + } ,\;{\text{step}}\,1} \right) $$

2 $$ { }\left[ {{\text{CuCl}}_{x} } \right]^{1 - x} + {\text{e}}^{ - } \to {\text{Cu}} + {\text{xCl}}^{ - } { }\left( {{ }0.7\;{\text{V vs}}.{ }{{{\text{Zn}}} \mathord{\left/ {\vphantom {{{\text{Zn}}} {{\text{Zn}}}}} \right. \kern-0pt} {{\text{Zn}}}}^{2 + } ,\;{\text{step}}\;2} \right) $$

Anode:

3 $$ {\text{Zn}} + 2{\text{Cl}}^{ - } \to {\text{ZnCl}}_{2} + 2{\text{e}}^{ - } $$

In voltage range of 0.4 ~ 1.6 V, the discharge capacity is 395 mAh g^−1^ based on Cu^II^ → Cu^I^ →  Cu^0^ transformation. More importantly, no Cu deposition on the Zn anode and no dendrite formation. With voltage window changed to 0.9–1.6 V, Cu only reacts with Cu^II^ →  Cu^I^, delivering 192 mAh g^−1^ discharge capacity. The results show excellent reaction/diffusion kinetics and improves the utilization rate of active materials.

A phase-separation electrolyte (PSE) is developed, which consists of an immiscible aqueous phase electrolyte and oil phase electrolyte. In this work, unlike the previous bi-phase aqueous/organic liquid electrolyte, the oil phase electrolyte acts as catholyte and the alkaline aqueous phase electrolyte acts as anolyte (Fig. 5h) [72].

First of all, due to the strong coordination between Zn^2+^ and OH^–^ (the stability constant of Zn(OH)_4_^2–^), Zn^2+^ ions are completely confined in the aqueous electrolyte. Through the study of inductively coupled plasma (ICP) and XRD, the Zn^2+^ ions hardly exist in the organic Py_14_TFSI-HFE component. So, the plating of zinc can only be processed in the aqueous phase component during charge, suggesting that the zinc dendrites cannot grow across the water–oil interface (Fig. 5i). Then, the cathode and anode of the battery after 10 cycles are observed by SEM, in which the obvious zinc dendrites and nanosheets can be observed for the zinc anode immersed in aqueous phase component of the PSE. In a sharp contrast, the images of zinc anode in the organic Py_14_TFSI-HFE component of the PSE demonstrate a pristine surface with no zinc plated, which confirms inference that the zinc dendrites cannot grow across the water–oil interface, thus completely avoiding the dendrite-induced short circuit.

#### PH-Decoupled Dual-Liquid-State Electrolytes

Conventional AMB are limited by the narrow ESW (~ 1.23 V) of water, PH-decoupled dual-liquid-state electrolytes are used acidic/alkaline electrolytes to provide a different electrochemical reaction environment for cathode and anode, extending the ESW of water for high-voltage aqueous batteries.

The high-voltage batteries require stable catholyte needs withstanding high oxidation potential, and the anolyte maintaining stability at low reduction potential. Barely aqueous electrolytes can simultaneously satisfy both high oxidation potential and low reduction potential. So, we need to look for asymmetric electrolytes that can meet the requirements of both cathode and anode.

**Fig. 6:**
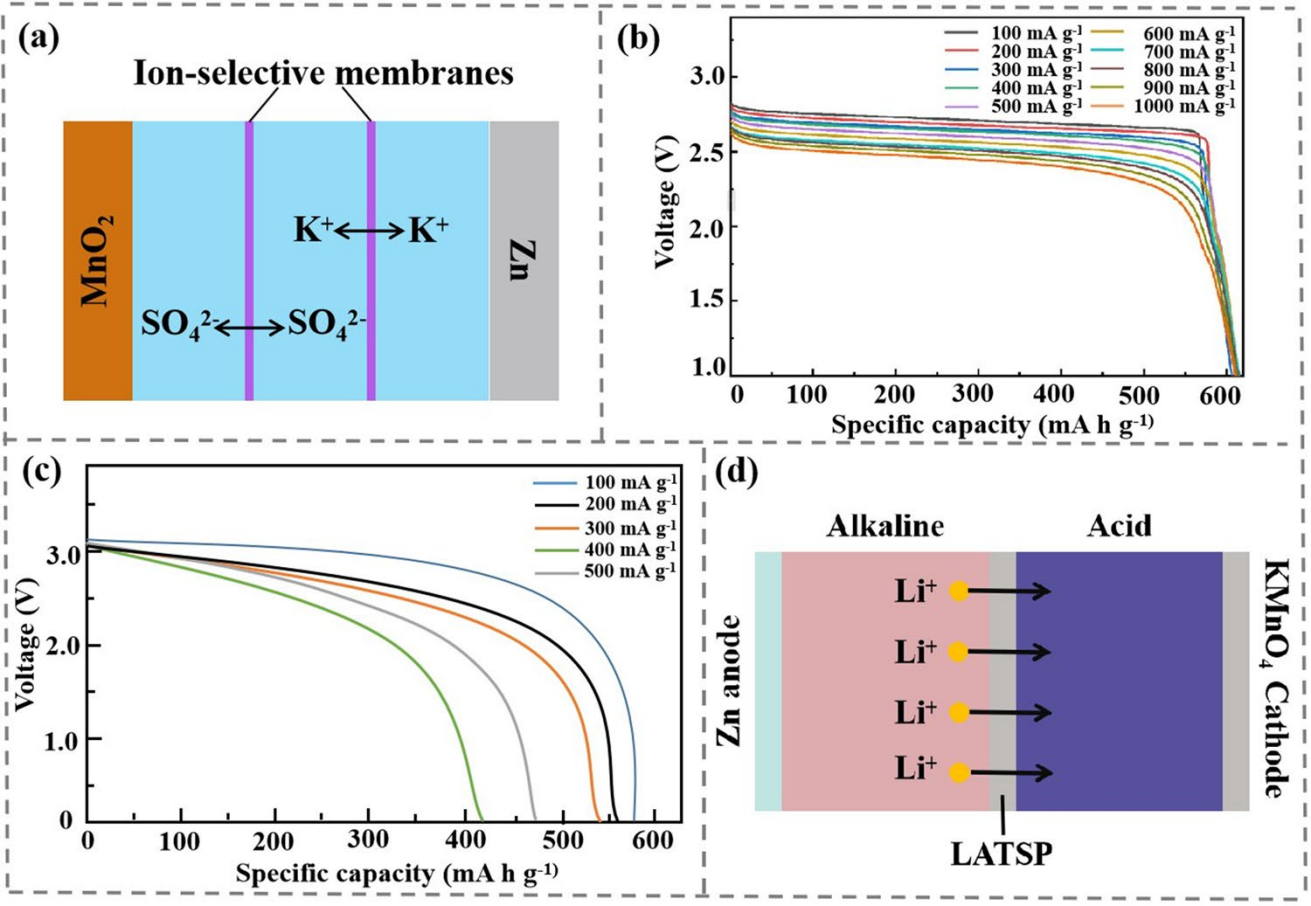
**a** Structure and operating principle of Zn//MnO_2_ battery with discoupled acid/alkaline electrolyte. Copyright 2020, Nature Publishing Group [24]. **b** Discharge curves for the DZMB at discharge current densities ranging from 100 to 1,000 mA g^−1^. Copyright 2020, Nature Publishing Group [24]. **c** Discharge curve of a conventional battery with the discharge current densities raised. **d** Structure of a high-voltage Zn//KMnO_4_ battery with decoupled acid/alkaline electrolytes [34]

AZMB’s low potential: Although aqueous electrolytes are safer and more environmentally friendly, low output voltage limits its application scenarios. Therefore, it is very necessary to develop high-voltage AMB. The electrochemical stability window of conventional homogeneous aqueous electrolyte is impossible larger than 3 V. In addition, alkaline Zn//MnO_2_ batteries have long been commercialized and widely used, but their operating voltage and rechargeability are limited due to operation in alkaline system. In the alkaline condition, the Zn anode can discharge stably, but the Mn cathode can produce irreversible by-products such as Mn(OH)_2_, Mn_2_O_3_ and Mn_3_O_4,_ which seriously reduce the deep discharge performance and cyclic stability [73], whereas the MnO_2_ cathode possesses good cyclic stability in neutral or mild acidic environment, which can meet the rapid reaction kinetics requirements of reversible ions (such as Zn^2+^, Li^+^) insertion/extraction. To prevent neutralization between acidic catholyte and alkaline anolyte, a neutral chamber was added to separate the catholyte, and a cation-exchange membrane is added to each catholyte (Fig. 6a). The cathode and anode reactions in the battery system are summarized as Eqs. (4–5):

4 $$ {\text{Zn}} + 4{\text{OH}}^{ - } \to {\text{Zn}}\left( {{\text{OH}}} \right)_{4}^{2 - } + 2{\text{e}}^{ - } { }\left( {E^{{\text{O}}} = - 1.199\;{\text{V vs}}.{\text{ SHE}}} \right) $$

5 $$ {\text{MnO}}_{2} + 4{\text{H}}^{ + } + 2{\text{e}}^{ - } \to {\text{Mn}}^{2 + } + 2{\text{H}}_{2} {\text{O }}\left( {E^{{\text{O}}} = { }1.224\;{\text{V vs}}.{\text{ SHE}}} \right) $$

Therefore, a decoupled asymmetric electrolyte is composed of 6 M KOH, 0.2 M ZnO and 5 mM vanillin as anolyte, was composed of 0.1 M K_2_SO_4_ as the neutral chamber [74], and 3 M H_2_SO_4_ and 0.1 M MnSO_4_ as catholyte [24]. Accordingly, the electrolytic Zn//MnO_2_ battery can achieves a high voltage of 2.83 V, much high than that of traditional Zn//MnO_2_ battery (1.5 V) [75]. The 3.33 Ah electrolytic Zn//MnO_2_ battery obtains an energy density of 90 Wh kg_cell_^−1^ at device level (three times the energy density of a lead-acid battery while the electrolyte is more environmentally friendly) [76]. In addition, the loss of battery capacity of Zn//MnO_2_ battery using decoupled electrolyte is only 2% after 200 h deep cycle, which indicates that MnO_2_ has been fully utilized and can maintain 100% capacity after various current density cycles.

Benefiting from high voltage and high utilization of MnO_2_, the discharge capacity and output voltage of Zn//MnO_2_ battery reaches 616 mAh g^−1^ at 100 mA g^−1^ and 2.71 V, respectively, much closer to 100% theoretical capacity (the double electron transfer process of Mn^4+^/Mn^2+^ is 617 mAh g^−1^). With the current density increased to 1,000 mA g^−1^, the output voltage of 2.44 V and the high specific capacity of 611 mAh g^−1^ can still be obtained. More importantly, electrolytic Zn//MnO_2_ battery maintains stable discharge voltages at different discharge depths (Fig. 6b). In sharp contrast, when the current density of traditional batteries increases, the stable voltage platform disappears, and the specific capacity rapidly declines (Fig. 6c), which severely limits the use of batteries.

Furthermore, an 3 V aqueous Zn//KMnO_4_ battery is developed based on acid-alkaline decoupled electrolytes [34]. Meanwhile, the ceramic lithium super-ionic conductor Li_1+x+y_Al_x_Ti_2_-_x_SiyP_3-y_O_12_, LATSP) thin film is used as a lithium ionic exchange membrane to separate the acidic and alkaline electrolytes. Consequently, H^+^ and OH^−^ cannot pass through the membrane to avoid neutralization reactions. In details, the Zn metal is used as anode, the 2 M KOH + 2 M LiOH is utilized as anolyte, and the cathode is KMnO_4_^−^ dissolved in 1M H_2_SO_4_ (Fig. 6d). The cathode and anode reactions in the battery system are summarized as Eqs. (6–7):

6 $$ 2{\text{KMnO}}_{4}^{ - 1} + 8{\text{H}}_{2} {\text{SO}}_{4} + 10{\text{Li}}^{ + } + 10{\text{e}}^{ - } \to 2{\text{MnSO}}_{4} + {\text{K}}_{2} {\text{SO}}_{4} + 5{\text{Li}}_{2} {\text{SO}}_{4} + 8{\text{H}}_{2} {\text{O}} $$

7 $$ {\text{Zn}} + 4{\text{OH}}^{ - } - 2{\text{e}}^{ - } \to {\text{ZnO}}_{2}^{2 - } + 2{\text{H}}_{2} {\text{O}} $$

The potential from MnO_4_^−^ to MnO_2_ is (1.69 V vs. SHE), and the theoretical discharge capacity of KMnO_4_ based on 3 e^−^ transfer is 904 mAh g^−1^. The catholyte is injected with 0.4 mg of KMnO_4_ every two hours to ensure that excess Zn is present in the Zn anode. The system can discharge for a long time, like a fuel cell. Considering the consumption of H^+^(H_2_SO_4_) and Li^+^(LiOH) for the reduction of KMnO_4_ and oxidation of Zn, real cathode capacity of KMnO_4_ is calculated to be 243.5 mAh g^−1^. As a result, the voltage of the Zn//KMnO_4_ cell reaches 3 V, and the theoretical energy density can reach 454 Wh kg^−1^.

### Bi-Phase Solid–Liquid Electrolytes

Compared with liquid electrolyte, quasi-solid–liquid electrolyte shows higher stability and smaller volume, which makes it have a broad application prospect in wearable devices. In quasi-solid electrolytes, it is often difficult for a homogeneous electrolyte to meet the requirements of rapid reaction kinetics of cathode and electrochemical stability of metal anode. Therefore, the utilization of bi-phase solid–liquid electrolyte can effectively solve many issues of both anode and cathode.

In bi-phase solid–liquid electrolytes, the liquid electrolyte is usually used on the cathode side, allowing the battery to achieve high ionic conductivity and good interface contact at cathode/electrolyte interface for fast cathode-side reaction kinetics requirements. However, the rapid realization of cathode-side reaction kinetics usually produces a lot of side reactions and cannot meet the needs of high chemical stability of anode side. Therefore, high-stability solid electrolyte is employed on the anode side for side-reaction-free metal electrolyte plating/stripping, which is not miscible with the liquid electrolyte on the cathode side. Normally, solid-state electrolyte as the anolyte can cut off the liquid-state electrolyte with anode contact. Therefore, the bi-phase electrolyte can fundamentally avoid HER and passivation reactions between the liquid-state electrolyte and anode.

**Fig. 7:**
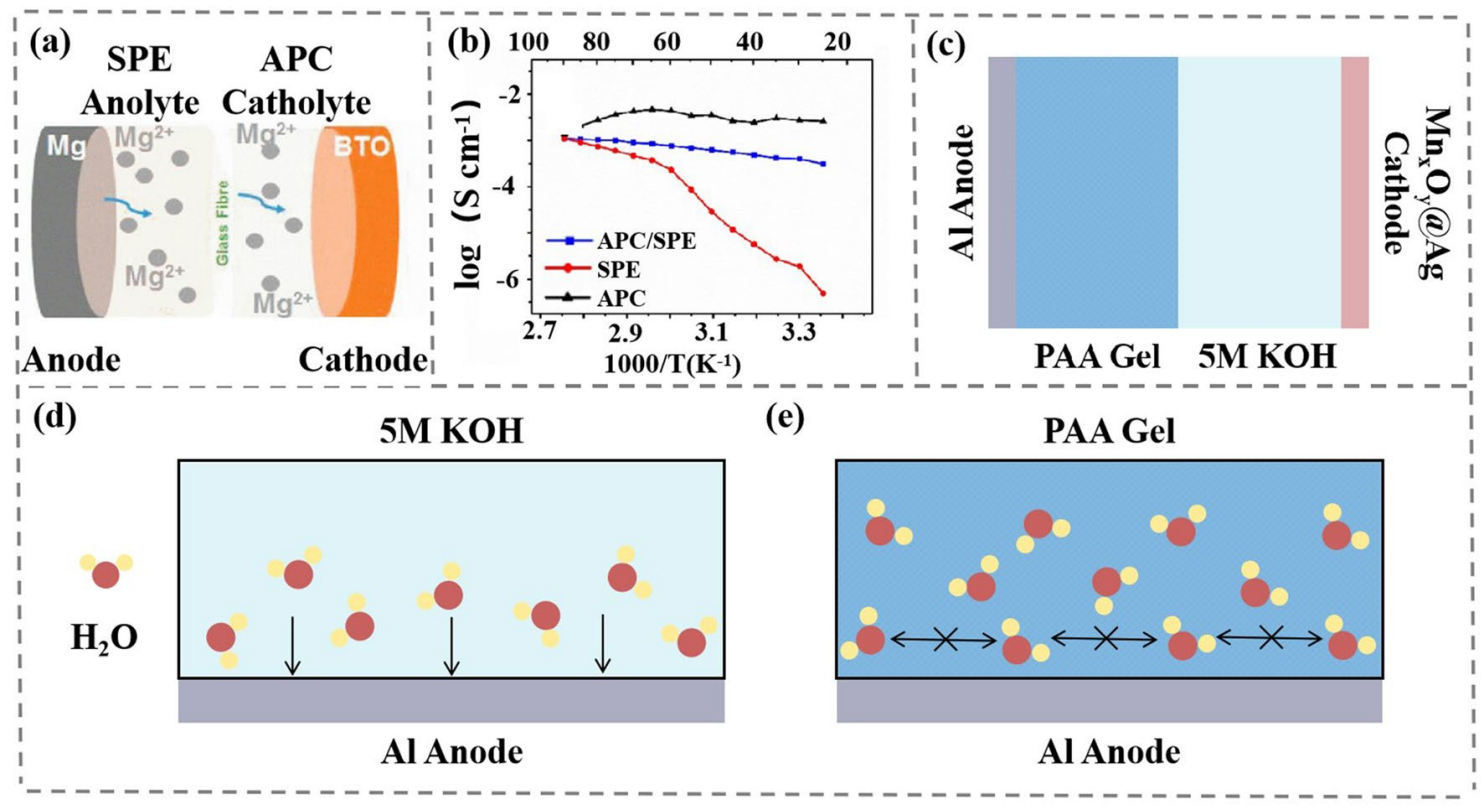
**a** Schematic diagram of Mg//APC/SPE//BTO battery. Copyright 2022, The Royal Society of Chemistry [42]. **b** Temperature-depended ionic conductivity for APC, SPE and APC/SPE electrolytes. Copyright 2022, The Royal Society of Chemistry [42]. **c** Basic structure of an aluminum-air battery with bi-phase solid-asymmetric electrolyte. **d-e** Aluminum anode is corroded by a large amount of free water in solution. In PAA gel, free water is fixed in the pores of PAA gel, and there is only a small amount of free water at the interface, so PAA gel protects the aluminum anode from water erosion

Rechargeable magnesium-ion battery (RMIB) has a high theoretical volumetric capacity (~ 3,833 vs. 2,062 mAh cm^−3^ for Li) and the low reduction potential (-2.4 V vs. SHE) [77, 78]. The kinetically sluggish Mg^2+^ insertion/extraction in the host lattice and cycle produces passivity film in the anode [79], which seriously slows down the transport kinetics of Mg^2+^ and shortens battery cyclic lifespan [80]. It is urgent to develop an electrolyte with high ion transfer kinetics and electrochemical compatibility with Mg metal anodes. Unlike LIB, Mg metal in contact with oxygen or a conventional electrolyte creates a “true passivation layer,” which limits plating/stripping process. Gel polymer electrolytes serve as a solid-state electrolyte, which has the advantages of high conductivity along with good mechanical strength, flexibility, good interface stability and better manufacturing integrity. It has the ability to reduce undesirable chemical reaction at the interface with Mg anode, thus the battery has lower internal resistance and longer cycle performance. A RMIB with a high Mg^2+^ conductivity (4.62 × 10^−4^ S cm^−1^ at 55 °C) is developed as shown in Fig. 7a [42]. Solid polymer electrolyte (SPE) cast from a mixture of PVDF, NMP, TEGDME and Mg(CF_3_SO_3_)_2_ exhibits high conductivity, high flexibility and excellent interface stability. Among them, the C = O polar group in TEGME can promote the decomposition of Mg(CF_3_SO_3_)_2_, thus speeding up ionic conductivity of Mg^2+^ at various temperature (Fig. 7b). SPE as anolyte can generate a stable semiconductor passivation layer, increasing the conduction rate of Mg^2+^ and inhibit dendrite growth and passivation layer generation. The 0.4 M Mg_2_Cl_3_^+^·AlPh_2_Cl_2_^−^/THF (APC) ionic liquid as catholyte enables the rapid insertion/extraction of Mg^2+^ from BTO without affecting the structure and chemical composition.

Aluminum-air batteries have been widely studied because of their high theoretical specific capacity (2980 mAh g^−1^), high volumetric capacity (8040 mAh cm^−3^ for Al vs 2046 mAh cm^−3^ for Li) and abundant reserves (7.73% of the Earth’s crust) [81]. However, aluminum-air batteries suffer from severe self-corrosion in alkaline aqueous solution, which seriously degrades the long cycle performance of batteries [82], leading to a large amount of waste of aluminum resources. People began to try to use solid electrolyte to solve the self-corrosion phenomenon of aluminum anode, but solid electrolyte cannot meet the fast reaction kinetics demand of air cathode, resulting in serious polarization of the battery [83], and the high theoretical specific capacity of aluminum battery cannot be fully reflected.

The redox reaction of cathode and anode of the battery during discharge is shown as follows [21]:

8 $$ {\text{O}}_{2} + 2{\text{H}}_{2} {\text{O}} + 4{\text{e}}^{ - } \to 4{\text{OH}}^{ - } { } E^{O} = { } + 0.4{\text{ V }} $$

9 $$ {\text{Al}} + 4{\text{OH}}^{ - } { } \to {\text{Al}}\left( {{\text{OH}}} \right)_{4}^{ - } + 3{\text{e}}^{ - } { }E^{{\text{O}}} = { } - 2.34{\text{V }} $$

When Al(OH)_4_^−^ reaches the saturation point, it precipitates from the electrode surface, and the chemical reaction occurs as follows [21]:

10 $$ {\text{Al}}\left( {{\text{OH}}} \right)_{4}^{ - } \to {\text{Al}}\left( {{\text{OH}}} \right)_{3} + {\text{OH}}^{ - } $$

The whole process of chemical reaction can be summarized as [21]:

11 $$ {\text{Al}} + 3/4{\text{O}}_{2} + 3/2{\text{H}}_{2} {\text{O}} \to {\text{Al}}\left( {{\text{OH}}} \right)_{3} { }E^{{\text{O}}} = { } + 2.74{\text{V }} $$

However, in open-circuit conditions, the corrosion-induced self-discharge of the active material prevails through following equation [21]:

12 $$ {\text{Al}} + 3{\text{H}}_{2} {\text{O}} + 3{\text{OH}}^{ - } \to {\text{Al}}\left( {{\text{OH}}} \right)_{4}^{ - } + 3/2{\text{H}}_{2} \uparrow { } $$

The theoretical voltage of the battery is 2.74 V, but in practice the voltage is usually limited to 1.2 ~ 1.6 V, which is ascribed to the generation of Al_2_O_3_ passivation layer and discharge product Al(OH)_3_. In addition, the parasitic water reduction bring about HER, leading to electrolyte consumption and battery swell.

An aluminum-air batteries is developed by an environmentally friendly bi-phase solid–liquid electrolyte for consisting of PAA hydrogel as anolyte and 4 M KOH alkaline solution as catholyte to simultaneously satisfy fast reaction kinetics and inhibit Al metal corrosion (Fig. 7c) [44]. The bi-phase electrolyte achieves a high ionic conductivity of 564.9 mS cm^−1^ and suppresses 88.6% hydrogen evolution, which significantly improves the power density and cyclic stability. This may be attributed to the fact that the hydrogel in the dual-electrolyte system absorbs a little bit of KOH solution, which increases the wettability of the Al anode surface and the generation of Al(OH)_3_ in Al anode is reduced. As a result, the battery has longer cycle performance (~ 14 times longer than that of the conventional alkaline electrolyte) and better power capability. Thanks to its porous structure, PAA gels lock H_2_O molecules in the pores, which greatly reduces the content of free water at the aluminum anode [84] (Fig. 7d-e). With inexpensive Mn_x_O_y_@Ag as a cathode and Al metal as anode, the Al-air battery system exhibits high specific capacity of 1563.6 mAh g^−1^, working voltage of 1.26 V at 20 mA cm^−2^ (200 mV higher than a single electrolyte) and a long cycle lifespan over 50 h (approximately 5 h for a single electrolyte).

### Asymmetric Quasi-Solid-State Electrolyte

Replacing conventional liquid electrolytes with quasi-solid-state electrolytes, the safety concerns of electrolyte leakage and flammability have been addressed. Nevertheless, a single solid-state electrolyte is incompatible with both the highly reductive anodes and oxidative high-voltage cathodes. Asymmetric solid-state electrolytes with more than one-layer electrolyte are capable of effectively tackle such issues by constructing a multiple layered-like structure.

The gel electrolyte has good mechanical stability, flexibility and ionic conductivity. The gel electrolyte containing liquid solution has a certain viscosity, so the interface between the gel electrolyte and the electrode has a low impedance and good interface contact. When the whole battery is deformed, the gel electrolyte can still cling to the electrode, which shows great research prospect in the field of flexible energy storage.

In the sub-section, we divided asymmetric quasi-solid electrolytes into asymmetric hydrogel-hydrogel electrolyte, asymmetric hydrogel-organgel electrolyte. Meanwhile, we discuss the design principles, synthetic methods, and electrochemical properties of asymmetric quasi-solid-state electrolytes.

#### Asymmetric Hydrogel–Hydrogel Electrolyte

Hydrogel electrolyte has a wide application prospect in the field of flexible wearable energy storage due to its high security, high ionic conductivity, and good interface compatibility. Hydrogel possesses three-dimensional (3D) network, whose mechanical strength can be further improved by adding polyols and inorganic particles (such as SiO_2_) via hydrogen bond and crystallinity. However, excess polyols and inorganic particles will highly increase the rigidity of the hydrogel and the flexibility will be weakened. Meanwhile, 3D structure enables hydrogen to contain abundant liquid solution, which has great influence on the electrochemical performance of battery. Hydrogels are prone to serious problems such as deformation and decreased ion conductivity after the loss of ionic liquid. The evaporation and freezing of ionic liquids at high or low temperatures are also urgent problems to be solved [85]. In addition, self-healing hydrogels can be repaired through hydrogen and ionic bonding, after being fractured by impact, torsion and other external forces.

**Fig. 8:**
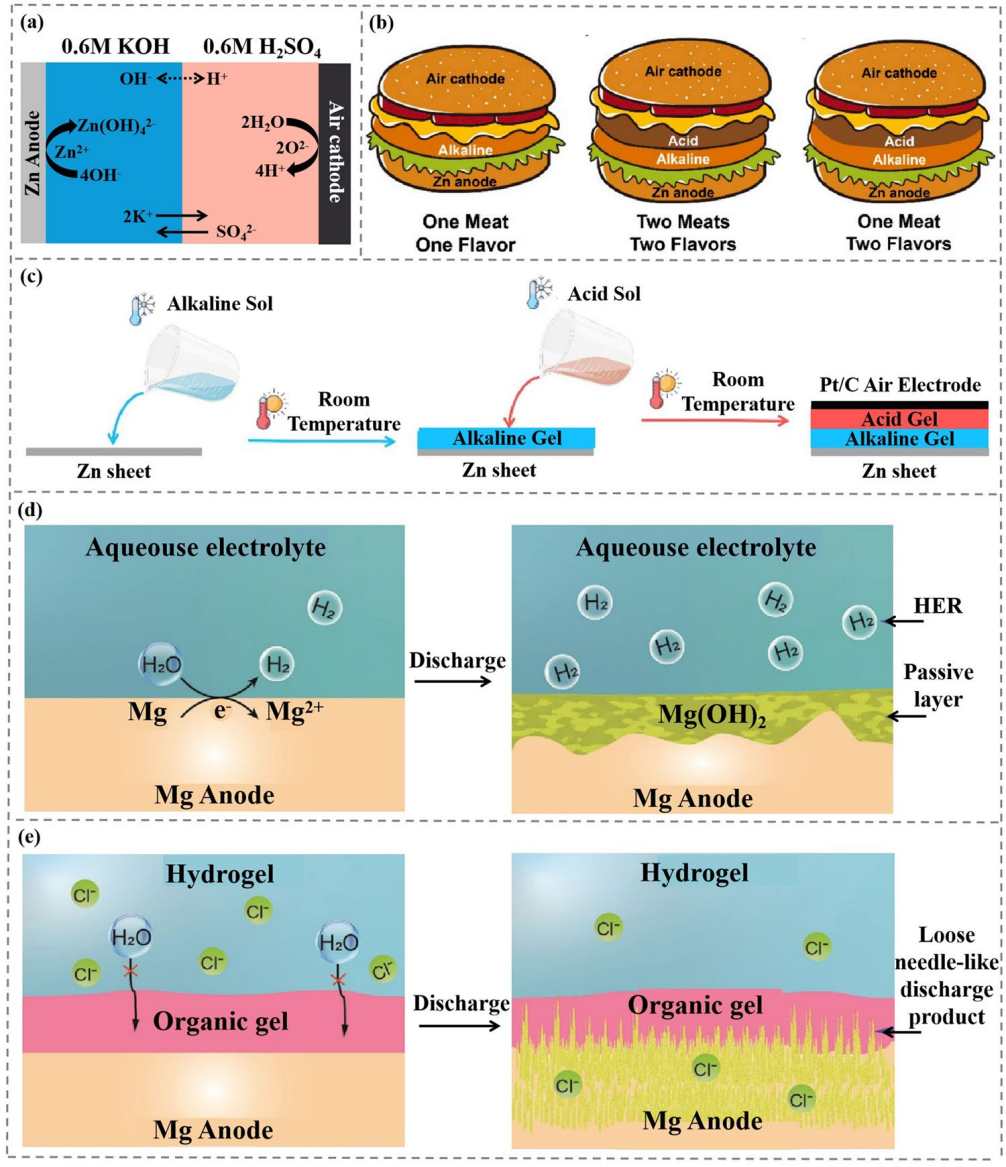
**a** Schematic illustration of the ion transport behavior within AAHE-based ZABs. Copyright 2022, Elsevier [25]. **b** Evolution process of sandwiches from (One Meat One Flavor) conventional structure → (Two Meats Two Flavors) recent double-meat structure with two flavors → (One Meat Two Flavors) novel “two flavors in one meat” structure, which demonstrated the design strategy of the flexible high-voltage ZAB with all-in-one and membrane structure AAHE. Copyright 2022, Elsevier [25]. **c** Fabrication process of the AAHE-based ZAB: Cold sol-state alkaline is poured on the Zn surface to form a conformal interface → After transforming to a gel-state alkaline at room temperature, cold sol-state acid is poured on it, followed by an air electrode on the top → return to room temperature for a gel-state acid. Copyright 2022, Elsevier [25]. Discharge process of the Mg-air batteries in the different electrolytes. **d** In conventional aqueous electrolytes, the H_2_ evolution side reaction occurs at the anode electrolyte interface. The side reaction and discharge process both produce a dense Mg(OH)_2_ passive layer, which separates the anode and electrolyte, leading to battery failure. Copyright 2021, Wiley–VCH [38]. **e** Proposed dual-layer gel electrolyte efficiently protects the Mg anode from corrosion and endows a loose needle-like discharge product, which keeps the discharge process active until the Mg anode is fully consumed. Copyright 2021, Wiley–VCH [38]

A Zn-Air cell (ZAB) is developed based on decoupled acid–base gel electrolytes using thermally reversible Pluronic®F127 hydrogel, where the acidic Pluronic®F127 hydrogel is used as catholyte while the alkaline Pluronic®F127 hydrogel is utilized as anolyte (Fig. 8a) [25]. It is noted thatpluronic®F127 hydrogel eliminates the utilization of expensive bipolar membranes, and meanwhile shows higher ionic conductivity, lower impedance and excellent adhesion during bending and other deformations [86, 87]. The structure of the traditional single electrolyte, the bi-layer electrolyte and the ZAB is shown in Fig. 8b. The cathode and anode reactions in the battery system are summarized as Eqs. (13) and (15). Equation (14) is the chemical reaction and standard potential of cathode in alkaline environment.

13 $$ {\text{Zn}} + 4{\text{OH}}^{ - } \to {\text{Zn}}\left( {{\text{OH}}} \right)_{4}^{2 - } + 2{\text{e}}^{ - } { } E^{{\text{O}}} = { } - 1.199{\text{V vs}}.{\text{ SHE }} $$

14 $$ {\text{O}}_{2} \left( {\text{g}} \right) + 2{\text{H}}_{2} {\text{O}} \to 4{\text{OH}}^{ - } { }E^{{\text{O}}} = { }0.401{\text{V vs}}.{\text{ SHE }}\left( {{\text{Alkaline}}\;\;{\text{ condition}}} \right){ } $$

15 $$ {\text{O}}_{2} \left( {\text{g}} \right) + 4{\text{H}}^{ + } + 4{\text{e}}^{ - } \to 2{\text{H}}_{2} {\text{O }}E^{{\text{O}}} = { }1.229{\text{V vs}}.{\text{ SHE }}\left( {{\text{Acidic}}\;\;{\text{ condition}}} \right){ } $$

It can be obviously observed that the potential of cathode in alkaline environment is much lower than that of cathode in acidic electrolyte. Therefore, replacing alkaline catholyte with acidic catholyte allows a battery to have a higher theoretical electromotive force, a higher energy density and a wider range of application scenarios.

Compared with catholyte under alkaline condition, the theoretical potential of catholyte under acidic condition is enlarged by about 0.8 V, which makes the ESW of ZAB greatly expanded. The ZAB achieves an electrochemical window of 2 V, far exceeding the 1.4 V of traditional ZAB [25]. At the same time, the ZAB exhibits high pressure stability for 37 h and a large area capacity of 1.35 mAh cm^−2^. Using Pluronic®F127 hydrogel’s unique sol–gel transition properties, an alkaline cold sol-state hydrogel can be poured onto a Zn anode and heated to room temperature to form a stable gel after waiting for the hydrogel and Zn anode to form a stable interface (Fig. 8c). An acidic cold sol-state hydrogel is then poured over anolyte and an air cathode is placed on it and heated to room temperature. Unprecedented film-free ZAB is prepared by simple operation. The sol–gel transition temperature of Pluronic®F127 hydrogel decreases after salt addition, further improving the stability of the system.

#### Asymmetric Hydrogel–Organgel Electrolyte

Compared with hydrogel electrolytes, organgel electrolytes can inhibit hydrogen evolution, corrosion and passivation layers better in the anode protection of metal air batteries. At the same time, the hydrogel electrolyte has good interfacial compatibility at the cathode, which can reduce the interfacial impedance and provide higher ionic conductivity.

Mg-Air battery (MAB) has a great advantage in reserves and price, with 2.08% Mg and 0.0065% Li in the earth’s crust [20]. When the discharge product is MgO, the theoretical volumetric density and a specific energy density of MAB are 14 kWh L^−1^ and 3.9 kWh kg^−1^, respectively, much higher than those of Li-air battery (8.0 and 3.4 kWh kg^−1^). MAB can work stably in neutral electrolyte, and Mg^2+^ has good biocompatibility. Therefore, MAB has a wide range of applications in flexible wearable devices and implantable electronic devices. However, MAB is prone to side reactions in water-based electrolytes and generates an insoluble dense passivation layer, which increases the internal resistance at the interface between Mg anode and electrolyte, resulting in reduced capacity and discharge voltage [88]. In the field of MAB, a double-layer asymmetric gel electrolyte with PEO as hydrogel and PAM as organgel [38] solves the problems of corrosion and dense passivation layer of Mg anode. The contact interface between PAM and PEO gel electrolytes is stable, and the ion conductivity remains basically constant. The interface between the asymmetric electrolytes has high interfacial compatibility and stability due to the force of hydrogen bond and has high ionic conductivity (6.52 mS cm^−1^). PEO as anolyte effectively prevents Mg from being corroded by H_2_O molecules (corrosion rate is 0.007 mg cm^−2^ h^−1^, 76 times lower than unprotected Mg). The corrosion reaction that usually occur is:

16 $$ {\text{Mg}} + 2{\text{H}}_{2} {\text{O}} \to {\text{Mg}}\left( {{\text{OH}}} \right)_{2} + {\text{H}}_{2} $$

The MAB is corroded after discharge in the aqueous electrolyte and the Mg anode protected by the double-layer gel electrolyte after discharge (Fig. 8d-e). With double-layer gel electrolyte, the fibrous MAB keeps the voltage basically constant and remains stabler more than 15 h when submerged in water**.** The system works with the small amount of oxygen dissolved in water and the MAB can still discharge in water without oxygen**,** suggesting potential application in implantable electronics. The MAB system has a specific capacity of up to 2,190 mAh g^−1^ (Mg anode utilization of 99.3%) and an energy density of up to 2,282 Wh kg^−1^ [20, 89].

After 48 h discharge, soft acicular discharge product is observed to grow vertically on the surface of Mg anode. It is generally believed that the acicular discharge product will affect the cycle life of the battery and even lead to short circuit of the battery. However, the acicular product is Mg_2_Cl(OH)_3_, which is different from the Mg (OH)_2_ commonly reported. The formation of the acicular discharge product is related to Cl^−^ and improves the discharge capacity of MAB.

## Challenge and Solution of Aqueous Batteries with Asymmetric Electrolyte

Due to the existence of different electrolyte/electrolyte interfaces in asymmetric electrolytes, this section focuses on the ion transport kinetic and interfacial compatibility.

Multivalent metal ion (e.g., Zn^2+^, Mg^2+^, Ca^2+^, Al^3+^) batteries are theoretically capable of carrying multiple electron transport, thus increasing charge carriers’ diffusion efficiency [90, 91]. However, multivalent metal ion battery shows long-term-seated issues of slow dynamics and difficult solvation/desolvation process at electrolyte/electrolyte and electrolyte/electrode interface [48].

### Slow Diffusion Kinetics

The desolvation of metal cations at the electrode/electrolyte phase/interface is the rate-determining step of the phase reaction in rechargeable batteries. Multivalent metal ions carry higher charge, theoretically allowing them to carry more charge at the same time, resulting in higher rate performance [92]. However, the charge density of polyvalent cations is much higher than that of univalent cations (e.g., the charge density of Li^+^ is 87 C mm^−3^, the charge density of Na^+^ is 36 C mm^−3^,the charge density of Mg^2+^ is 205 C mm^−3^, the charge density of Zn^2+^ is 181 C mm^−3^ and that of Al^3+^ is 770 C mm^−3^) [93]. This indicates that multivalent cations exhibit higher solvation energy, which reduces ion kinetics at the interface, resulting in slow diffusion kinetics and higher energy barriers [48]. Calcium accounts for about 4.1% of all elements in the Earth’s crust, about 1,000 times that of lithium. The redox potential of Ca^2+^/Ca is − 2.87 V vs. SHE. Therefore, calcium-ion batteries (CIBs) can provide a higher operating voltage, comparable to lithium-ion batteries. The volumetric and gravimetric capacities of Ca metal are 2,073 and 1,337 mAh g^−1^. However, the insertion kinetics of multivalent ions into hosting structures is generally considered sluggish than monovalent Li ions [94]. In multivalent ions, the radius of Ca^2+^, Mg^2+^, Al^3+^ and Zn^2+^ are 0.99 Å, 0.86 Å, 0.57 Å and 0.76 Å, respectively. In CIBs, calcium ions have slow kinetics at the electrode due to their large radius. To solve the sluggish dynamic and the interactions with other elements of Ca^2+^, CIBs usually need to operate at moderate temperature (above 100 °C [19]) or even high temperatures (above 500 °C even 850 °C). The increase in the operating temperature of CIBs solves the kinetics problem of Ca^2+^ plating/stripping at the anode. However, due to the increase in working temperature, Ca^2+^ is more likely to be unevenly distributed when planted at the anode, resulting in dendrite growth. Electrolyte corrosion of the electrode will also be more serious, which will affect the cycle life and capacity of CIBs.

In traditional aqueous electrolytes, the charge density and ionic radius of various cations are different, and the solvation structure of cations formed in the electrolyte is also different. In aqueous dilute solution electrolytes, metal cations usually form a two-layer solvation structure with water, the first solvation shell is relatively tight, while the second solvation shell is relatively sparse. An asymmetric electrolyte is designed as Zn(OTf)_2_ as anolyte and LiTFSI as catholyte [43], and Zn^2+^ is transported in anolyte and Li^+^ is transported in catholyte. As usual, in the first solvation shell, Zn^2+^ forms the solvated structure of Zn[(H_2_O)_6_]^2+^ with water, and Li^+^ forms the solvated structure of Li[(H_2_O)_4_]^+^ with water. In asymmetric electrolyte systems, the rate performance of the battery is limited by electrolytes with slow solvation/desolvation rates. The solvation/desolvation rate of organic electrolytes is usually slower than that of aqueous electrolytes, so the rate performance of the battery is easily limited by organic electrolytes [33].

**Fig. 9:**
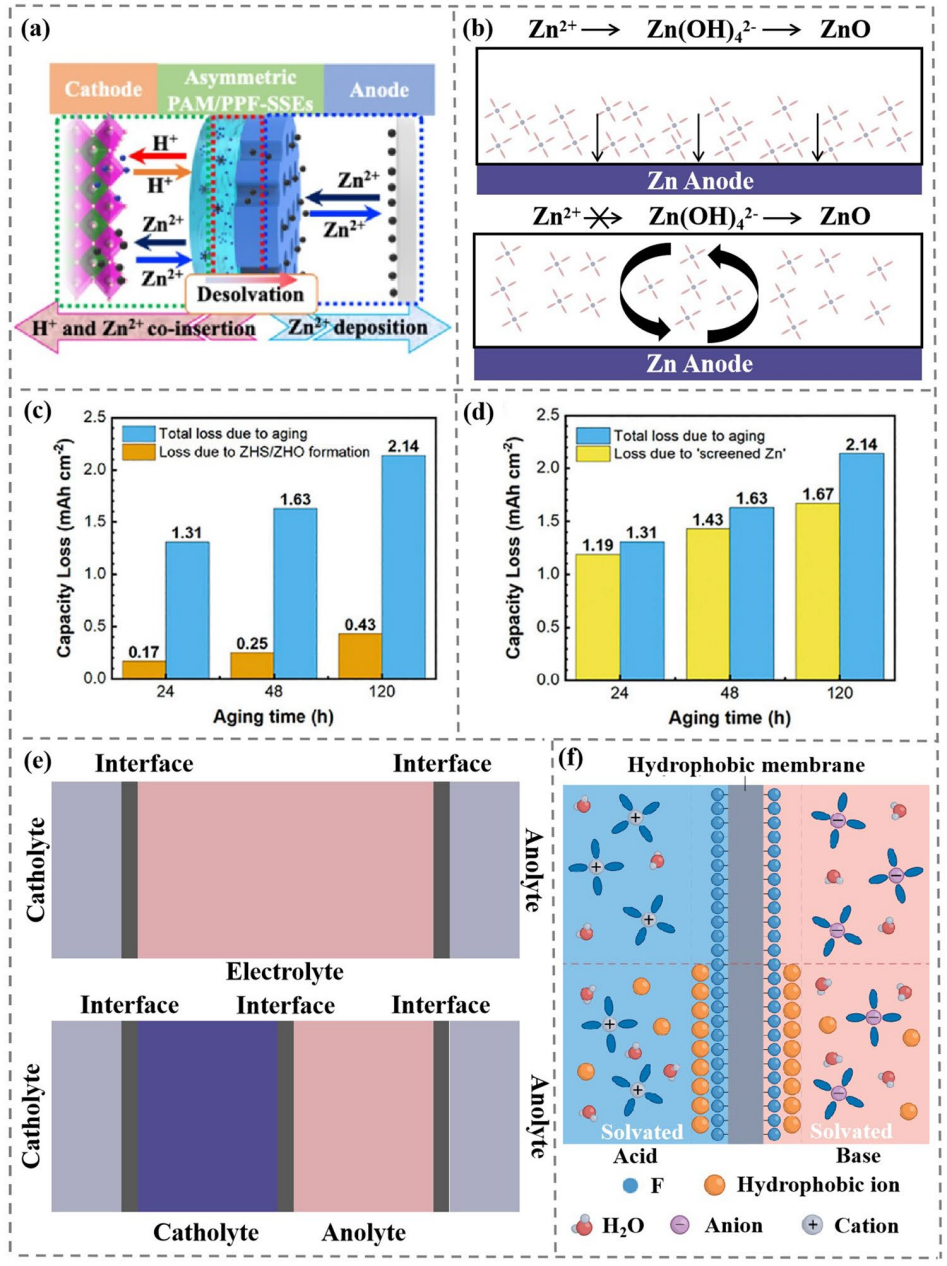
**a** Schematic illustration of asymmetric electrolyte design (including one-layer PPFs-SSEs and one-layer PAM hydrogel electrolyte) [45]. Copyright 2023, Nature Publishing Group. **b** In the flowing anolyte, the formation of Zn (OH))_2_ by Zn^2+^ is avoided and thus the formation of ZnO passivation layer is avoided. **c** Comparison of the Zn anode capacity loss for different aging times due to ZHS/ZHO formation, measured using online MS from a sealed vial cell, and the total anode loss, measured electrochemically from an equivalent coin cell. Copyright 2023, Elsevier [97]. **d** Capacity loss with different aging times due to screened Zn formation (measured by MS of H_2_ evolved from H_2_SO_4_ titrated onto anodes retrieved from the cycled coin cells) and the total anode loss (measured electrochemically from the coin cells). Copyright 2023, Elsevier [97]. **e** Conventional electrolytes have two electrode/electrolyte interfaces, while asymmetric electrolyte has an additional catholyte and anolyte interface. **f** A hydrophobic membrane can selectively adsorb and transport hydroponic ions, the purple anions and gray cations in panel F refer in particular to OH^−^ and H^+^. Copyright 2022, Nature Publishing Group [60]

An asymmetric electrolyte combining an inorganic solid electrolyte with a hydrogel electrolyte was designed to satisfy both the reversible inhibition of Zn plating/stripping on the anode side and the rapid insertion/extraction of Zn^2+^ on the cathode side [45, 95]. The design diagram is shown in Fig. 9a. To meet the needs of fast reaction kinetics, the Zn//MnO_2_ batteries are constructed using 1 M Zn(OTf)_2_ in distilled water as aqueous electrolytes and 1M Zn(OTf)_2_ in trimethyl phosphate (TMP) as non-aqueous electrolytes as reference [45]. The battery with aqueous electrolyte delivers a specific capacity close to theoretical capacity of 300 mAh g^−1^, while with non-aqueous electrolyte, it only shows 191.1 mAh g^−1^. The same phenomenon is observed in Zn//V_2_O_5_ battery systems. In addition, the capacity of Zn//V_2_O_5_ batteries decays to 49.7% initial capacity after only 200 cycles with non-aqueous electrolyte. In comparison, the battery gives 93.9% capacity retention with an aqueous electrolyte. These results prove that using aqueous electrolytes as catholyte can increase the diffusion rate of Zn^2+^ and reduce the energy barrier of Zn^2+^ as it crosses the catholyte/cathode interface. When the battery is charged and discharged, Zn^2+^ forms a Zn[(H_2_O)_6_]^2+^ solvation structure with water molecules in catholyte, and water acts as a lubricant to lower the diffusion barrier of Zn^2+^, which can provide a higher capacity. The Zn^2+^ changes its solvation structure at the catholyte-anolyte interface, and Zn^2+^ forms solvation structure in anolyte with TMP. The organic electrolyte can isolate the contact between water molecules and the zinc anode, thereby eliminating the hydrogen evolution reaction between the zinc anode and water, which greatly improves the cycle life and capacity retention rate of the battery. However, Zn^2+^ does not form solvated structures at the same rate as water and TMP. The transport rate and solvation/desolvation rate of Zn^2+^ in aqueous electrolyte are both faster than that in non-aqueous electrolyte. As a result, the ion conduction rate of the electrolyte as a whole is limited by the ion conduction rate of the non-aqueous electrolyte. This will also limit the battery’s rate performance and power density.

### Parasitic Side Reactions

Although alkaline electrolyte is widely used for aqueous batteries, the metal anodes suffer from corrosion and rampant hydrogen evolution in alkaline conditions. The discharge product Zn(OH)_4_^2−^ reaches the upper limit of solubility, and ZnO passivation layer is produced on Zn metal anode [96]. The passivation layer is formed in two steps in traditional static batteries: firstly, the Zn anode forms a soft porous layered structure in a supersaturated solution. When the electrolyte changes from saturated state to unsaturated state, the porous layer becomes a hard and dense layered structure adsorbed on the Zn anode, which reduces battery capacity and lowers ion conductivity.

In alkaline electrolyte, the Zn/ZnO potential is -1.249 V vs. SHE and the hydrogen evolution potential are -0.828 V vs SHE. According to the principle of thermodynamics, the HER is inhibited in the alkaline electrolyte. However, after the formation of ZnO passivation layer, the Zn potential increases and the HER becomes more intense. To solve the problems of corrosion and HER, we can use a stirring battery to allow the anolyte to flow and disrupt the conditions for the passivation layer to form (Fig. 9b) [40].

Gas precipitation from battery is the main reason (over 80%) for the irreversible depletion of Zn anode in the weak acidic electrolyte [97]. Screening effect occurs with gases accumulation on Zn anode surface, which displaces the electrolyte on the surface and prevents the anode and electrolyte from conducting normal ion transport. Zn(OH)_2_/Zn_4_SO_4_(OH)_6_·*x*H_2_O (ZHO/ZHS) contributes less than 20% of the capacity loss while “screened Zn” leads to 80% of the capacity loss (Fig. 9c-d). To reduce capacity loss in charge/discharge process, a simple degassing process can be performed on anode and the close-touch anode/electrolyte interface is restored to execute ion transport, increasing the coulombic efficiency from 47.8 to 81.6%.

Although the above methods can effectively alleviate the degradation of Zn anode in aqueous electrolyte, it is difficult to change the thermodynamic instability of Zn anode [98]. Therefore, the use of organic electrolyte as anolyte fundamentally prevents the contact between water and Zn anode to avoid corrosion of Zn anode by active water molecules.

Tripropylene glycol (TG) was added to the aqueous electrolyte, breaking the original hydrogen bond network and changing the solvation structure of Zn^2+^ and water [99]. With the addition of TG to the solvation structure of Zn^2+^, the distance between Zn^2+^ and Zn anode during nucleation increases, thus increasing the energy barrier of Zn deposition. TG will be adsorbed on the surface of the anode after it reaches the Zn anode, thus reducing the contact between water and the Zn anode and inhibiting HER, passivation and other side reactions. A similar idea can also be used in the design of asymmetric electrolyte. Organic electrolyte collects on the anode to isolate the contact between water and anode, and fundamentally prevent water corrosion of anode [100].

### Interfacial Non-Compatibility and Resistance

Multilayer asymmetric electrolytes possess more interfaces than conventional electrolytes (Fig. 9e). We should not only pay attention to interfacial impedance, ionic conductivity and interface compatibility at the solid–liquid transition interface of electrolyte/electrode, but also interface compatibility between catholyte and anolyte. In non-membrane liquid/liquid electrolytes, the non-membrane structure can reduce the interface impedance and allow AMB to have a higher power density. The degree of immiscibility and compatibility of ionic solution are improved by selecting two electrolytes with large polarization difference. However, in high-voltage AMB using decoupled acidic catholyte and alkaline anolyte, H^+^ and OH^−^ react slowly over long cycling tests, which rapidly deteriorates the energy density and cycling stability due to PH change.

To achieve long-term lifespan, expensive BPM with selective ion transport characteristics is often used to separate catholyte from anolyte. The catalysts in BPM can decompose H_2_O to form H^+^ and OH^−^ to restore the acidity and alkalescence of catholyte and anolyte. Theoretically, H^+^ and OH^−^ are both hydrophilic ions [101], which are adsorbed on the surface of IEM with strong hydrophobic substances (such as F^−^, OTF^−^ and TFSI^−^). Thus, BPM can effectively prevent the transmembrane transport of H^+^ and OH^−^ for their neutralization reactions at the interface (Fig. 9f) [60].

Compared with the liquid electrolyte, the quasi-solid electrolyte/liquid electrolyte interface and the quasi-solid electrolyte/quasi-solid electrolyte interface have better stability. Quasi-solid electrolyte can confine ions inside the gel to a certain extent and can effectively inhibit H^+^ and OH^−^ reactions in the acid-alkaline asymmetric electrolyte system, thus improving the long cycle performance of the battery. In the aqueous-organic liquid-state asymmetric electrolytes system, the quasi-solid electrolyte can effectively avoid the miscibility of anolyte and catholyte. However, this condition also results in an increase in impedance at the anolyte/catholyte interface, resulting in a decrease in the ionic conductivity of the overall electrolyte. At the same time, the ion conduction rate of the quasi-solid electrolyte is also lower than that of the liquid electrolyte, which will weaken the battery’s rate performance and power density.

## Characterization Technique

Different from the traditional homogeneous electrolytes, the interface compatibility between catholyte and anolyte should be studied as well as the interface of electrodes/electrolytes. Relevant studies can be carried out by typical characterization methods, such as scanning electron microscope (SEM), transmission electron microscope (TEM), X-ray photoelectron spectroscopy (XPS), Raman spectroscopy (Raman), X-ray diffraction (XRD), in situ optical microscopy (OM), molecular dynamics (MD) and density function theory (DFT). In this section, we divide the scope of characterization into anolyte/catholyte interface and electrolyte interior, electrolyte/electrode interface and electrode surface. The applications of various characterization methods are discussed respectively.

### Anolyte/Catholyte Interface and Electrolyte Interior

**Fig. 10:**
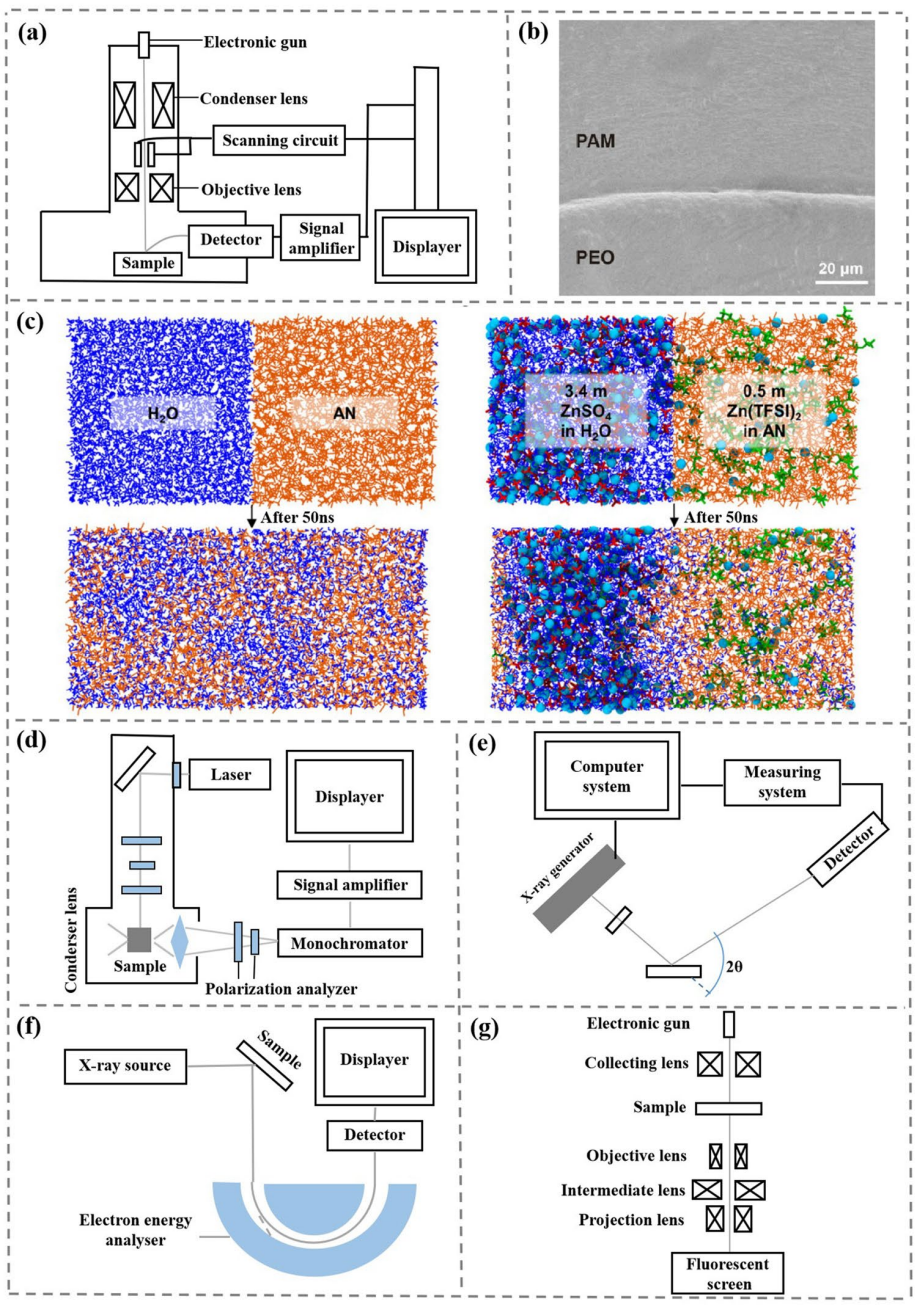
**a** Structure and schematic diagram of SEM.** b** SEM image of the dual-layer gel electrolyte [38]. Copyright 2021, Wiley–VCH. **c** MD of aqueous asymmetric electrolyte. H_2_O, AN, Zn^2+^, SO_4_^2−^, and TFSI^−^ molecules are depicted as blue, orange, cyan, red, and green colors, respectively [36]. Copyright 2022, The Royal Society of Chemistry. **d** Structure and schematic diagram of Raman. **e** Structure and schematic diagram of XRD. **f** Structure and schematic diagram of XPS. **g** Structure and schematic diagram of TEM

Compared to conventional single electrolytes, asymmetrical electrolytes have an additional anolyte/catholyte interface between catholyte and anolyte. The study of this interface is very important in the study of asymmetric electrolytes. When studying the anolyte/catholyte interface, it is common to use a microscope for initial observation in the gel-state electrolyte system. By observing whether there is a clear dividing line at the anolyte/catholyte interface, the close contact and interface compatibility can be preliminarily judged. There is a close-touch interface between gel-state electrolyte with no obvious boundary, suggesting good interface compatibility (Fig. 10a-b) [38].

For decoupled liquid asymmetric electrolyte, due to the large fluidity of liquid solutions, the anolyte/catholyte interface is difficult to characterize by traditional technologies. Recently, with the development of computer technology, theoretical calculation has been widely used in battery field, which can more intuitively and accurately show the distribution of various molecules and ions in liquid electrolytes and the coordination structure of molecules/ions in electrochemical reactions. The kinetic parameters of electron transfer can be analyzed accurately. MD records the motion state and position of each atom at different periods in 3D space, then calculates the interaction force between each molecule based on the position and motion state, and further analyzes the subsequent position and motion state of each molecule. Therefore, MD can be used to study molecular diffusion and solvation structure changes at the interface of anolyte/catholyte of liquid asymmetric electrolytes. MD can also be used to observe the uniform distribution of electrolyte within a liquid state. For example, pure AN and pure H_2_O are fully fused at 50 ns, and AN and H_2_O show obvious interface stratification after the addition of Zn(TFSI)_2_ and ZnSO_4_ (Fig. 10c) [36]. The molecules in the interior of anolyte and catholyte are also evenly distributed and did not accumulate in small areas.

### Electrolyte/Electrode Interface and Electrode Surface

HER and OER at the electrode/electrolyte can be clearly observed by using *in situ* optical microscopy. By observing the amount of bubbles in aqueous electrolyte and asymmetric electrolyte of Zn anode, it can be deduced that asymmetric electrolyte can better inhibit HER [36]. Since the Raman scattering of aqueous solution is relatively weak, Raman is suitable for studying the molecular structure of solvent molecules in aqueous solution and can be used for qualitative analysis of material changes in electrochemical reactions (Fig. 10d). Raman can also be used at the anode/electrolyte interface to compare an asymmetric electrolyte system with a conventional electrolyte. There is a large amount of ZnO and Zn(OH)_2_ in hydrogel, while Zn anode with asymmetric electrolyte showing dendrite-free and side reactions-free plating/stripping process with highly reversibility [45].

When studying the electrode/electrolyte interface, the change of material composition of SEI can be studied using XRD and XPS (Fig. 10e-f) [24]. With discharging progress, the Bragg peak of MnO_2_ does not change, and the intensity gradually weakens until it disappears completely. With charging progress, the Bragg peak of MnO_2_ shows the opposite phenomenon, indicating that the MnO_2_ of the cathode has good reversibility. XPS further confirms the high reversibility judgment. The peak position of Mn *2P* at 642.2 eV remains unchanged with the discharge and the peak intensity gradually weakened until completely disappeared when the discharge is completed. TEM can reach a resolution of 0.1 nm and can observe the micro and nanostructure of atoms, so TEM is very suitable for observing the different morphology of crystals during phase transition (Fig. 10g). The morphology and crystal structure of MnO_2_ cathode before and after charge and discharge are observed by TEM when studying atomic deposition at MnO_2_ cathode [24]. After charging, it can be seen that MnO_2_ atoms are deposited on the surface of the electrode, and MnO_2_ disappears after deep discharging process. This intuitively shows that the discharge process of the battery is related to the gradual dissolution of MnO_2_, and the charging process is related to the electrochemical deposition of MnO_2_. While studying the MnO_2_ cathode of ZnǀǀMn batteries, DFT calculations are carried out to reveal the intrinsic modulation effect of Ni dopant on MnO_2_ during electrolysis process [102]. After the introduction of high charge density Ni, the electron density of O decreases, which causes the energy level of O *p* to shift upward. The whole reaction process takes place on a surface with a lower potential energy. This promotes charge transfer, which promotes catalyze electrolytic kinetics and lower overpotential.

In addition to the above characterization methods, there are still many characterization methods that can be applied to the study of asymmetric electrolyte system batteries. For example, atomic force microscopy (AFM) can be used to observe the process of Zn nucleation, especially the dendrite growth caused by the tip effect. Fourier transform infrared spectroscopy (FT-IR) and nuclear magnetic resonance (NMR) can obtain information about the molecular structure or intermolecular interactions of electrolytes, and it can play an important role in the study of solvation structure in electrolytes. In the future study of asymmetric electrolytes, more *in situ* characterization methods and theoretical calculation models need to be developed.

## Conclusions and Perspectives

This review presents the concept of asymmetric electrolyte with multi-layer structure in aqueous multivalent metal ion batteries. Liquid-state aqueous electrolytes, liquid-state organic electrolytes, hydrogel and organgel are employed as cathode or anolyte to assembled with cathode and anode, respectively, for high-voltage and high-cyclic stability batteries. Battery with asymmetric electrolyte can simultaneously meet the requirements of rapid reaction kinetics of cathode and thermodynamic stability of anode. Meanwhile, the asymmetric electrolyte shows wider electrochemical stability window and high ion conductivity. We conclude that battery with asymmetric electrolyte structure has outstanding performance in the following aspects.

1. Thermodynamics and dynamics requirements: Metal anode is thermodynamically unstable in aqueous electrolytes, which is easy to cause anode corrosion and dendrite growth, deteriorating delivered capacity and cyclic stability. The organic electrolyte acting as anolyte can avoid the formation of by-product and improve thermodynamic stability of metal anode. However, organic electrolytes cause serious erosion and low diffusion kinetics of cathode, leading crystal structure collapse and active materials dissolution. Therefore, the selection of bi-phase aqueous/organic electrolyte can simultaneously meet both thermodynamic stability of metal anode and fast diffusion kinetic requirements of AMB.
2. Electrode protection: The electrolyte as the ionic conductor between anode and cathode, connecting anode and cathode to form a loop. At present, many battery systems improve the performance of the electrolyte to achieve higher electrode stability. However, many strategies that promote the formation of *in situ* SEI by adding additives to the electrolyte do not protect the electrode well. Using the solid-state anolyte with high mechanical strength can effectively reduce dendrite growth, and the hydrophobic anolyte can effectively mitigate the passivation reaction of anodes by reducing the free water in anodes. At the cathode, aqueous electrolyte can protect the structural stability of the cathode while meeting the fast kinetic requirements.
3. Wider electrochemical stability window: Different from traditional single electrolyte structure, asymmetric electrolyte can provide two different electrochemical reaction chambers for anode and cathode, respectively. As a result, the overall electrolyte has a higher *E*_g_ (*e.g.,* The *E*_g_ of liquid water increases from ~ 1.23 V in a single environment to > 2 V in a decoupled acidic/alkaline asymmetric electrolyte. Likewise, bi-phase aqueous/organic electrolyte and bi-phase solid–liquid electrolyte systems are also possible to choose different electrolyte environments to make higher reducing potential at the electrode, this allows the battery to have a higher OCV.

Although asymmetric electrolytes revolutionize the development of electrolytes and significantly enhance battery performance, there is still spaces for improvement. Based on the research, we summarize the future direction of asymmetric electrolyte as follows:

1. Higher energy density: AMB are often limited by the narrow electrochemical stability window of a homogeneous electrolyte. In comparison, the PH-decoupled asymmetric electrolytes separate anolyte and catholyte by IEM and neutral chamber to avoid volume reduction due to neutralized reaction between H^+^ and OH^−^. However, IEM and neutral chamber cannot increase the specific capacity while increasing the volume and weight of batteries. To solve the neutralization reaction between H^+^ and OH^−^, quasi-solid-state electrolytes is an effective approach to confine H^+^ and OH^−^ in gel. It is also expected that electrolyte can provide additional capacities will be developed in aqueous/organic asymmetric electrolyte system.
2. Low ionic conductivity: Although aqueous electrolyte exhibits high ionic conductivity and fast diffusion kinetics. However, the utilization of sluggish organic/quasi-solid-state electrolytes layer and anolyte/catholyte interface result in ion transport hysteresis, which bring about poor rate capability and low power density of batteries. In addition, the solvation structure changes at the interface of anolyte and catholyte in dual-layer liquid-state electrolyte also need further study to develop rapid desolvation and solvation interfaces. Therefore, it is necessary to develop high-compatibility and high ion conductivity interface for high-performance batteries.

At the anolyte/catholyte interface, only a small amount of work has been done to investigate the energy and kinetic processes required for the solvation change process. Metal ions form solvated structures at different rates in different electrolytes, resulting in poorly matched metal ion transport rates on both sides of the anolyte/catholyte interface, which also affects the overall ionic conduction rate of the electrolyte.
